# Parametric resonance and chaos in a duffing-type oscillator with periodic inertia modulation

**DOI:** 10.1038/s41598-026-45221-w

**Published:** 2026-05-20

**Authors:** Mohamed El-Borhamy, Arafa A. Nasef, Abdel-Fattah Attia, Ismail Sobhy

**Affiliations:** 1 Department of Engineering Mathematics and Physics, Faculty of Engineering, Tanta University, 31527 Tanta, Egypt; 2 Department of Engineering Mathematics and Physics, Faculty of Engineering, Kafrelsheikh University, 33516 Kafr El Sheikh, Egypt; 3 Department of Computer and Systems Engineering, Faculty of Engineering, Kafrelsheikh University, 33516 Kafr El Sheikh, Egypt

**Keywords:** El Borhamy–Rashad–Sobhy equation, Harmonic balance analysis, Multiple scales analysis, Bifurcation and chaos, Piezoelectric energy harvester, Engineering, Mathematics and computing, Physics

## Abstract

This study presents a unified analytical–numerical framework for the El Borhamy–Rashad–Sobhy equation with Duffing-type nonlinearity, augmented by an external harmonic forcing term. The used application in the study is motivated by the electromechanical dynamics of salient-pole synchronous machines. Starting from an energy-based formulation, the harmonic balance method is used to obtain closed-form frequency-response relations. The local stability of the periodic orbit is further quantified via a Floquet-based test derived from the linear variational equation, yielding stability-classified frequency-response curves. While the method of multiple scales captures primary, superharmonic, and subharmonic resonance conditions and the associated stability boundaries are analyzed. Beyond the weakly nonlinear regime, numerical bifurcation analysis is performed to trace a period-doubling route to chaos, supported by Poincaré sections, the largest Lyapunov exponent, and time-series diagnostics. The system is further coupled with linear and nonlinear piezoelectric energy-harvesting branches, to demonstrate that, the large-amplitude responses enhance harvested power and the cubic stiffness can be tuned for bandwidth optimization. Overall, the results bridge perturbation theory with nonlinear time-domain diagnostics, providing design-relevant insights for broadband energy harvesters and electromechanical systems operating under parametric excitation and external forcing.

## Introduction

Vibratory phenomena in mechanical, electromechanical, and structural systems often exhibit responses that cannot be captured by simple linear models^1,2^. Beyond purely sinusoidal steady states, nonlinear oscillators may display amplitude-dependent frequency shifts, jump phenomena, and bifurcations, with possible transitions to chaos as control parameters vary^3^. Foundational perturbation treatments of these effects are presented in^4^, whereas broader nonlinear-dynamics viewpoints linking qualitative changes in invariant sets and attractors to engineering responses are summarized in^5^. This attractor-based perspective is particularly useful for interpreting routes to irregular motion and chaotic responses under parameter variation.

A precise understanding of such nonlinear effects is crucial for engineering design in which reliability and robustness are essential^6^. For example, nonlinear vibration and targeted energy transfer concepts relevant to structural and mechanical systems are surveyed in^7^. In structural dynamics practice, experimentally oriented identification, validation, and interpretation issues in nonlinear regimes are emphasized in^8^. From a modern modeling viewpoint, updated theory-to-practice treatments of nonlinear vibrations are consolidated in^9^, while recent perspectives highlight how nonlinearities reshape performance envelopes and motivate uncertainty-aware design workflows in engineering applications.

A canonical framework for studying nonlinear vibration is the Duffing oscillator, characterized by its cubic stiffness nonlinearity. Owing to its conceptual simplicity and representative dynamics, it has long served as a benchmark for theoretical analysis and experimental validation^10^, and it is also widely used to illustrate bifurcations and routes to complex motion in nonlinear dynamics^11^. In practical settings, however, oscillators are seldom isolated: damping, external forcing, and parametric modulation often play significant roles and can reshape stability margins and operating envelopes^12,13^. Representative examples include beams with variable tip masses, where parameter variation can induce backbone bending and multivalued responses^14^, electrostatically actuated micro-resonators where nonlinearities constrain stable operation and complicate the frequency response^15,16^, and broadband-excited energy harvesters where nonlinear stiffness is exploited to widen the effective bandwidth. Nonlinear phenomena also arise in rotating machinery and active magnetic bearing systems, where whirling motion and rub/impact interactions can generate strongly nonlinear responses and complex stability limits^17^.

Parametric excitation, in which system parameters vary periodically in time, further enriches the dynamic response. Classical Mathieu-type models show that even weak modulation can generate instability tongues in parameter space^18^. When combined with cubic stiffness, parametric systems can exhibit coupled primary, superharmonic, and subharmonic resonances and may undergo bifurcations leading to irregular and chaotic responses. Recent extensions have addressed electromechanical resonators with time-varying parameters and saturation-induced nonlinearities^19^. In related electro-mechanical settings, second-order multiple-scales analysis have been used to resolve subtle effects such as vibration center shift under steady and unsteady loads, and to design mitigation strategies^20^. Moreover, electromechanical realizations with time-varying inductive effects provide additional motivation for parametric modeling in machine-like dynamics^21^.

Two analytical techniques are particularly effective for investigating these systems. The harmonic balance method (HBM) assumes a truncated Fourier expansion and yields closed-form frequency-response relations, making it well suited for capturing backbone bending and jump phenomena^22–24^. The method of multiple scales (MMS) introduces slow time scales to derive amplitude-phase modulation equations and associated stability boundaries^25,26^. These perturbation tools remain especially useful when complemented by numerical continuation and time-domain diagnostics in regimes where weak-order assumptions no longer hold. In addition, HB-based approaches coupled with Floquet-type stability checks have proven effective in controlled vibration settings, supporting the use of HB as a practical tool for steady-response prediction and stability assessment^23^.

While weakly nonlinear perturbation tools (e.g., multiple scales) are effective near resonant regimes, practical rotating-machinery configurations-such as rotor-AMB systems-can also exhibit nonlinear whirl responses and intermittent rub/impact interactions, as reported for a 16-pole constant-stiffness rotor-AMB model^17^. Recent comparative work on rotor active magnetic bearing (AMB) rotors shows that the choice between fixed and adjustable surplus-current strategies can substantially modify the steady nonlinear response level, the propensity for impact (rub/contact) events, and the associated stability characteristics, with these effects examined across variations in rotor eccentricity and spin-speed detuning^27^. Such evidence supports complementing perturbation-based resonance tools with direct time-integration and nonlinear-dynamics diagnostics when targeting strongly nonlinear regimes.

Building on this foundation, a linear prototype known as the El Borhamy–Rashad–Sobhy equation has been proposed as a benchmark parametrically excited model and used to construct stability charts and transition boundaries^28,29^. Subsequent studies have reported additional variants and application-motivated formulations, including extended stability analysis and electromechanical interpretations^30–32^. The El Borhamy–Rashad–Sobhy equation, which can be expressed in normalized form as:

1 $$\begin{aligned} \bigl (1+h\cos \varOmega x\bigr )y'' + \frac{Q}{\alpha } y' + \frac{1}{\alpha ^2}y = 0, \qquad |h|<1. \end{aligned}$$

Here, *h* denotes the modulation depth, *Q* is a damping parameter, and $\alpha$ represents a frequency-scaling parameter. This prototype captures essential features of systems with periodically varying gain or loss, variable-inductance RLC circuits, and AC machine dynamics, and it provides a convenient reference point for assessing parametric-instability regions. In addition, Popov-based stability conditions and nonlinear control designs have recently been developed for Duffing-type extensions of the El Borhamy–Rashad–Sobhy dynamics, providing a stability-guaranteed control viewpoint that complements modeling and Floquet analysis^32^.

**Fig. 1:**
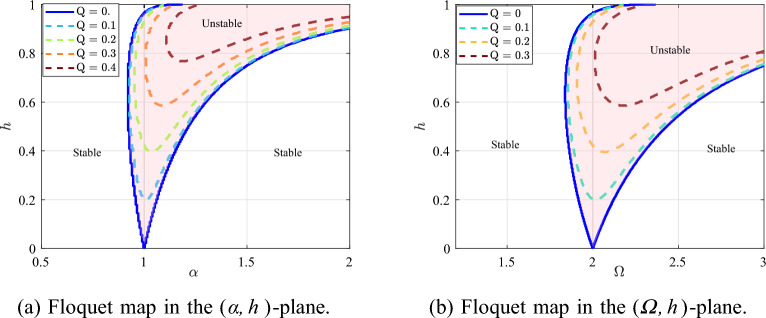
Floquet stability boundaries for different values of *Q*.

As a baseline, Fig. 1 plots representative Floquet stability boundaries in the $(\alpha ,h)$- and $(\varOmega ,h)$-planes for several values of *Q*. The unstable region expands with increasing modulation depth *h* and is progressively suppressed as the damping *Q* increases.

In this work, we generalize Eq. (1) by introducing cubic stiffness nonlinearity and external harmonic forcing, resulting in a Duffing-type system with harmonically modulated inertia. This nonlinear extension enables a systematic exploration of nonlinear resonances, multi-stability, bifurcations, and chaos. Furthermore, we incorporate a coupled energy-harvesting branch-both linear and nonlinear-to investigate how resonance interactions and complex responses can be leveraged to enhance broadband power capture.

The objectives of this study are therefore twofold: (i) to establish a unified analytical-numerical framework for characterizing the dynamics of a parametrically excited Duffing-type oscillator and (ii) to demonstrate its relevance as a design platform for optimized nonlinear energy harvesters. To this end, we derive the governing equation from an electromechanical model of a salient-pole synchronous machine, apply HBM and MMS to obtain analytical predictions and validate them against direct time-domain integration in their regime, and then extend the analysis using numerical bifurcation and time-series diagnostics to characterize strongly nonlinear and chaotic responses.

Despite the recent interest in Eq. (1) and its use in constructing Floquet stability charts, most available studies remain focused on the linear benchmark and on delineating parametric-instability boundaries. A clear gap persists in formulating a fully nonlinear Duffing-type extension with simultaneous inertia modulation and external forcing, and in establishing a unified link between MMS/HB predictions and numerical diagnostics to capture multistability, jump phenomena, and possible transitions to chaos. Moreover, Eq. (1) motivated energy-harvesting investigations still lack verification-driven connections between complex nonlinear responses and quantitatively validated harvested-power gains.

To address these limitations, this work proposes a forced Duffing-type El Borhamy–Rashad–Sobhy extension with harmonically modulated inertia, derives it from a salient-pole synchronous-machine electromechanical model, and combines matched-order MMS and HBM analysis with numerical validation and nonlinear-dynamics tools. In addition, we integrate linear and nonlinear harvesting branches and verify the harvested-power formulation by cross-checking period-averaged numerical power against harmonic-based estimates, thereby demonstrating how nonlinear/chaotic responses can enhance broadband power capture.

The main contributions of this work are summarized as follows:

- Extension of the El Borhamy–Rashad–Sobhy linear model to a fully nonlinear Duffing-type oscillator with parametric inertia modulation and harmonic forcing.
- Derivation of a consistent electromechanical formulation linking salient-pole synchronous machine physics to the normalized governing equation.
- Analytical characterization of the frequency-response curves and resonance conditions using HB and MMS, offering complementary insights.
- Validation of the weak-order analytical predictions (HB/MMS) against direct time-domain integration in the near-resonance regime, and extension to the strongly nonlinear domain via numerical bifurcation diagrams, Poincaré sections, and the largest Lyapunov exponent, thereby documenting a period-doubling route to chaos.
- Demonstration that nonlinear and chaotic responses can significantly enhance the harvested power in both linear and nonlinear energy-harvesting branches.

The remainder of the paper is organized as follows: Section 'Salient-pole synchronous machine' derives the electromechanical model and governing equation. Section 'Harmonic balance analysis' applies the HBM to approximate frequency-response curves. Section 'Multiple scales analysis' develops the MMS formulation to capture amplitude-phase modulation and parametric resonance. Section 'Comparison between harmonic balance and multiplescales' benchmarks the HBM and MMS primary-resonance predictions against direct numerical time integration. Section 'Bifurcation and chaotic response' reports numerical bifurcation and chaos analyses. Section 'Piezoelectric energy harvesting: linear and nonlinearmodeling' presents the energy-harvesting application and discusses the effect of nonlinear responses on power output. Section 'Verification of harvested-power formulation' verifies the consistency of the harvested-power formulation by comparing period-averaged numerical power to harmonic-based estimates. Finally, Section 'The conclusion' summarizes the main findings and highlights potential directions for future research.

## Salient-pole synchronous machine

**Fig. 2:**
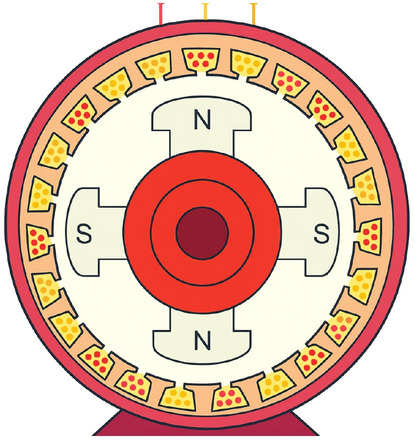
Schematic of a salient-pole synchronous machine highlighting air-gap saliency and field excitation.

The system studied in this paper is physically motivated by the electromechanical dynamics of a salient-pole synchronous machine (see Fig. 2). Such machines are widely employed in power generation and in high-torque, low-speed applications. Due to the projecting poles, the air-gap permeance varies periodically with the rotor position $\theta (t)$, which leads to a stator inductance that is periodically modulated^19,21^. This inductance modulation introduces a natural parametric effect in the electrical subsystem. In addition, magnetic saturation gives rise to a nonlinear flux-current relationship that can be approximated by a cubic term, thereby introducing Duffing-type nonlinearity in a reduced mechanical model. Together, these mechanisms make the salient-pole machine a natural platform for studying parametrically excited nonlinear oscillations.

Consider a single stator phase with flux linkage modeled as

2 $$\begin{aligned} \lambda (t)=L\bigl (\theta (t)\bigr )\,i(t)+\gamma \,i^{3}(t), \end{aligned}$$

where $i(t)=\dot{q}(t)$ is the phase current, *q*(*t*) is the electric charge, and $\gamma >0$ is an effective saturation coefficient. Applying Kirchhoff’s voltage law gives

3 $$\begin{aligned} V_s(t)=R\,i(t)+\frac{d\lambda (t)}{dt}. \end{aligned}$$

Using Eq. (2) and the product rule,

4 $$\begin{aligned} \frac{d\lambda }{dt}&=\frac{d}{dt}\Big (L(\theta )\,i\Big )+\frac{d}{dt}\Big (\gamma i^{3}\Big )\nonumber \\&=L(\theta )\,\dot{i}+\dot{L}(\theta )\,i+3\gamma i^{2}\dot{i}, \end{aligned}$$

with

5 $$\begin{aligned} \dot{L}(\theta )=\frac{dL}{d\theta }\,\dot{\theta }. \end{aligned}$$

Substituting Eq. (4) into Eq. (3) yields

6 $$\begin{aligned} V_s(t)=R\,i(t)+\Big (L(\theta (t))+3\gamma i^{2}(t)\Big )\dot{i}(t)+\dot{L}(\theta (t))\,i(t). \end{aligned}$$

For small saliency $(0<h\ll 1)$, the inductance can be expressed in the standard weak-modulation form

7 $$\begin{aligned} L(\theta )=L_0\Big (1+h\cos \big (\varOmega \theta \big )\Big ), \qquad 0<h\ll 1, \end{aligned}$$

so that $\dot{L}(\theta )=O(h)\,\dot{\theta }$. In the reduced-order modeling adopted here, the term $\dot{L}(\theta )\,i$ acts as a higher-order electromechanical feedback contribution compared with the inertial electrical term $L(\theta )\dot{i}$ and is neglected at leading order, yielding

8 $$\begin{aligned} V_s(t)\approx R\,i(t)+\Big (L(\theta (t))+3\gamma i^{2}(t)\Big )\dot{i}(t). \end{aligned}$$

Equation (8) makes explicit that the electrical dynamics are parametrically influenced by $\theta (t)$ through $L(\theta )$, while saturation introduces an amplitude-dependent nonlinear contribution.

The electromagnetic torque arises from the dependence of magnetic co-energy on rotor position. For a single-phase variable-inductance model, the co-energy is

9 $$\begin{aligned} W'=\tfrac{1}{2} L(\theta )i^2 \end{aligned}$$

and therefore

10 $$\begin{aligned} T_{\textrm{em}}(t)=\frac{\partial W'}{\partial \theta } =\frac{1}{2}\,i^{2}(t)\,\frac{dL(\theta )}{d\theta }. \end{aligned}$$

This relation clarifies the coupling: $\theta (t)$ modulates $L(\theta )$ in the electrical equation, while the electrical current *i*(*t*) generates mechanical actuation through the saliency gradient $dL/d\theta$.

In synchronous operation, the current contains a dominant harmonic component at the electrical excitation frequency. Accordingly, the torque in Eq. (10) admits a Fourier representation, and we retain its fundamental harmonic to obtain a tractable single-degree-of-freedom mechanical model suitable for subsequent perturbation analyses:

11 $$\begin{aligned} T_{\textrm{em}}(t)\approx F_{0}\cos (\varOmega t), \end{aligned}$$

where $F_{0}$ is an effective torque amplitude determined by the excitation level and machine parameters, and $\varOmega$ is the dominant excitation frequency.

Accordingly, the model is formulated about a synchronous operating point in which the electromagnetic torque admits a dominant harmonic component at the excitation frequency [Eq. (11)]. Thus, $\theta (t)$ represents the angular ripple about the mean rotation, i.e., $\theta (t)=\theta _s+\widetilde{\theta }(t)$.

The rotor torque balance is modeled as a Duffing-type oscillator about this operating point, including viscous damping, linear stiffness, and a cubic restoring term capturing local nonlinearities (e.g., saturation-induced stiffness effects and/or geometric nonlinearities under reduction):

12 $$\begin{aligned} J\,\ddot{\theta }(t)+D_{\theta }\dot{\theta }(t)+k_{\theta }\theta (t)+k_{3}\theta ^{3}(t)=T_{\textrm{em}}(t). \end{aligned}$$

In addition to producing a harmonic torque component, saliency-induced periodic terms in the reduced electromechanical coupling lead, after averaging/reduction and normalization, to a parametric modulation of the coefficient multiplying $\ddot{\theta }$. We model this effect as a harmonically modulated effective inertia

13 $$\begin{aligned} J(t)=J\bigl (1+h\cos (\varOmega t)\bigr ), \qquad 0<h\ll 1, \end{aligned}$$

so that Eqs. (12) and (11) yield the reduced forced parametric model

14 $$\begin{aligned} J\bigl (1+h\cos (\varOmega t)\bigr )\ddot{\theta }(t)+D_{\theta }\dot{\theta }(t)+k_{\theta }\theta (t)+k_{3}\theta ^{3}(t) =F_{0}\cos (\varOmega t). \end{aligned}$$

This form preserves (i) harmonic forcing through the dominant torque component and (ii) weak parametric modulation through the leading periodic contribution represented by *h*.

Introduce the dimensionless time $x=\varOmega t$, such that

15 $$\begin{aligned} \frac{d}{dt}=\varOmega \frac{d}{dx}, \qquad \frac{d^{2}}{dt^{2}}=\varOmega ^{2}\frac{d^{2}}{dx^{2}}, \end{aligned}$$

and define $y(x)=\theta (t)$. Substituting into Eq. (14) and dividing by $J\varOmega ^{2}$ gives

16 $$\begin{aligned} \bigl (1+h\cos (\varOmega x)\bigr )y''+\frac{D_{\theta }}{J\varOmega }\,y' +\frac{k_{\theta }}{J\varOmega ^{2}}\,y+\frac{k_{3}}{J\varOmega ^{2}}\,y^{3} =\frac{F_{0}}{J\varOmega ^{2}}\cos (\varOmega x). \end{aligned}$$

Let $\varOmega _{0}=\sqrt{k_{\theta }/J}$ be the linear natural frequency and introduce the dimensionless parameters

17 $$\begin{aligned} \alpha =\frac{\varOmega }{\varOmega _{0}}, \qquad Q=\frac{D_{\theta }}{\varOmega _{0}J}, \qquad \beta =\frac{k_{3}}{k_{\theta }}, \qquad F=\frac{F_{0}}{J\varOmega ^{2}}. \end{aligned}$$

Using Eq. (17), we obtain

18 $$\begin{aligned} \frac{D_{\theta }}{J\varOmega }=\frac{Q}{\alpha }, \qquad \frac{k_{\theta }}{J\varOmega ^{2}}=\frac{\varOmega _{0}^{2}}{\varOmega ^{2}}=\frac{1}{\alpha ^{2}}, \qquad \frac{k_{3}}{J\varOmega ^{2}}=\frac{k_{3}}{k_{\theta }}\frac{k_{\theta }}{J\varOmega ^{2}}=\frac{\beta }{\alpha ^{2}}. \end{aligned}$$

Therefore, Eq. (16) reduces to El Borhamy–Rashad–Sobhy equation with Duffing-type nonlinearity, augmented by an external harmonic forcing term:

19 $$\begin{aligned} \bigl (1+h\cos (\varOmega x)\bigr )y'' +\frac{Q}{\alpha }y' +\frac{1}{\alpha ^{2}}y +\frac{\beta }{\alpha ^{2}}y^{3} =F\cos (\varOmega x). \end{aligned}$$

This nonlinear extension of Eq. (1) provides a physically motivated and analytically tractable model for the stability, resonance, bifurcation, and energy-harvesting investigations developed in the following sections.

## Harmonic balance analysis

In this section, we derive a closed-form approximation of the steady-state periodic response of the periodically forced system Eq. (19) using the Harmonic Balance method^33–35^. The central objective is to establish explicit algebraic relations that connect the response amplitude and phase to the excitation frequency and the governing parameters, thereby enabling an efficient characterization of resonance features and parameter-dependent nonlinear trends.

Starting from the governing Eq. (19), we assume the first harmonic approximation

20 $$\begin{aligned} y(x)= & A_{0}+\sum _{n=1}^{\infty }\left[ A_n \cos (n\varOmega x) + B_n \sin (n\varOmega x)\right] \approx A_{0}+A_1\cos (\varOmega x) + B_1\sin (\varOmega x), \end{aligned}$$

21 $$\begin{aligned} R= & \sqrt{A_1^2 + B_1^2}, \end{aligned}$$

where *R* is the oscillation amplitude. Its derivatives are

$$y' = \varOmega \bigl (-A_1\sin \varOmega x + B_1\cos \varOmega x\bigr ), \qquad y'' = -\varOmega ^2\bigl (A_1\cos \varOmega x + B_1\sin \varOmega x\bigr ).$$

For the cubic term, only the zero-harmonic and fundamental harmonic components are retained:

22 $$\begin{aligned} y^3 \;\approx \; \Bigl (A_0^3+\frac{3}{2}A_0 R^2\Bigr ) +3\Bigl (A_0^2+\frac{R^2}{4}\Bigr )\bigl (A_1\cos \varOmega x + B_1\sin \varOmega x\bigr ). \end{aligned}$$

Substituting into Eq. (19) and using $\cos ^2(\varOmega x)=\tfrac{1}{2}(1+\cos 2\varOmega x)$ and $\sin (\varOmega x)\cos (\varOmega x)=\tfrac{1}{2}\sin 2\varOmega x$, we obtain

23 $$\begin{aligned}&\Biggl [\Bigl (-\varOmega ^2+\frac{1}{\alpha ^2}+\frac{3\beta }{\alpha ^2}\Bigl (A_0^2+\frac{R^2}{4}\Bigr )\Bigr )A_1 +\frac{Q\varOmega }{\alpha }B_1 - F\Biggr ]\cos (\varOmega x) \nonumber \\&\quad + \Biggl [\Bigl (-\varOmega ^2+\frac{1}{\alpha ^2}+\frac{3\beta }{\alpha ^2}\Bigl (A_0^2+\frac{R^2}{4}\Bigr )\Bigr )B_1 -\frac{Q\varOmega }{\alpha }A_1\Biggr ]\sin (\varOmega x) \nonumber \\&\quad + \Biggl [\frac{1}{\alpha ^2}A_0+\frac{\beta }{\alpha ^2}\Bigl (A_0^3+\frac{3}{2}A_0R^2\Bigr ) -\frac{h\varOmega ^2}{2}A_1\Biggr ] \nonumber \\&\quad -\frac{h\varOmega ^2}{2}A_1\cos (2\varOmega x) -\frac{h\varOmega ^2}{2}B_1\sin (2\varOmega x) + HOH =0. \end{aligned}$$

Here *HOH* denotes the neglected higher-order harmonics (e.g., $2\varOmega , 3\varOmega , \ldots ).$ Therefore, equating the coefficients of 1, $\cos (\varOmega x)$ and $\sin (\varOmega x)$ yields

24 $$\begin{aligned} \frac{1}{\alpha ^2}A_0+\frac{\beta }{\alpha ^2}\Bigl (A_0^3+\frac{3}{2}A_0R^2\Bigr ) -\frac{h\varOmega ^2}{2}A_1&= 0, \end{aligned}$$

25 $$\begin{aligned} \Bigl (-\varOmega ^2+\frac{1}{\alpha ^2}+\frac{3\beta }{\alpha ^2}\Bigl (A_0^2+\frac{R^2}{4}\Bigr )\Bigr )A_1 +\frac{Q\varOmega }{\alpha }B_1&= F, \end{aligned}$$

26 $$\begin{aligned} \Bigl (-\varOmega ^2+\frac{1}{\alpha ^2}+\frac{3\beta }{\alpha ^2}\Bigl (A_0^2+\frac{R^2}{4}\Bigr )\Bigr )B_1 -\frac{Q\varOmega }{\alpha }A_1&= 0. \end{aligned}$$

from which elimination of the phase yields the nonlinear frequency-response relation

27 $$\begin{aligned} \Bigl (-\varOmega ^2 + \frac{1}{\alpha ^2} + \frac{3\beta }{\alpha ^2}\Bigl (A_0^2+\frac{R^2}{4}\Bigr )\Bigr )^2 + \Bigl (\frac{Q}{\alpha }\varOmega \Bigr )^2 = \Bigl (\frac{F}{R}\Bigr )^2. \end{aligned}$$

Equation (27) relates the amplitude *R* to the excitation frequency $\varOmega$, damping *Q*, stiffness nonlinearity $\beta$, and forcing amplitude *F*.

**Fig. 3:**
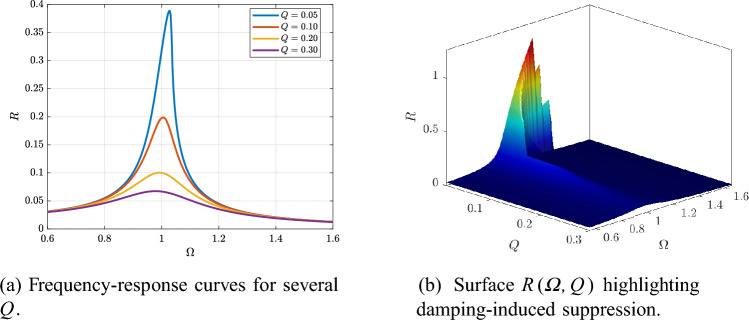
Effect of damping *Q* on the HB-predicted response at fixed $\beta$ and *F*.

Figure 3a shows the HB frequency-response curves $R(\varOmega )$ for different damping values *Q* (with $\beta$ and *F* fixed). Increasing *Q* lowers and broadens the resonance peak, confirming the expected dissipative suppression of the steady-state response. Figure 3b summarizes this trend through the surface $R(\varOmega ,Q)$, where the high-amplitude resonant ridge at small *Q* progressively collapses as *Q* increases.

**Fig. 4:**
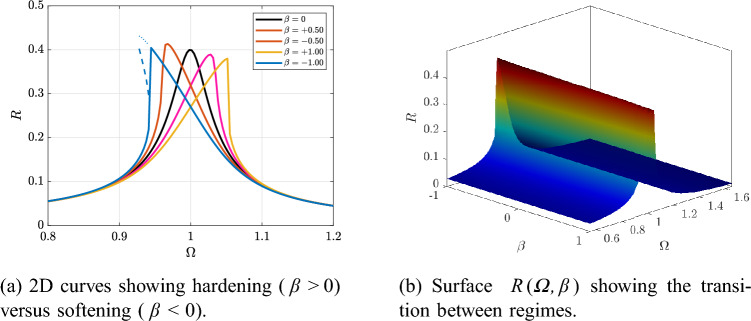
Effect of cubic stiffness $\beta$ on the frequency-response at fixed *Q* and *F*.

Figure 4a illustrates the effect of the cubic stiffness $\beta$. The surface $R(\varOmega ,\beta )$ in Fig. 4b makes this continuous transition clear, as the resonant ridge shifts across $\varOmega$ when $\beta$ changes sign.

**Fig. 5:**
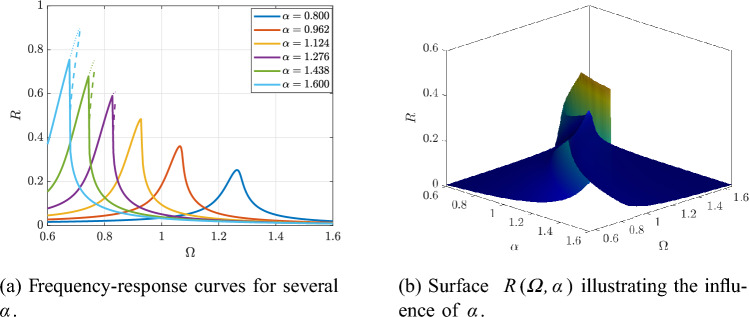
Effect of the inertia-scaling parameter $\alpha$ on the HB-predicted response at fixed $(Q,\beta ,F)$.

Figure 5a shows that varying the inertia-scaling parameter $\alpha$ shifts the resonance location and redistributes the response amplitude by modifying the effective linear frequency and detuning. This global tuning effect is compactly captured by the surface $R(\varOmega ,\alpha )$ in Fig. 5b, where the resonance ridge drifts in $\varOmega$ as $\alpha$ varies.

**Fig. 6:**
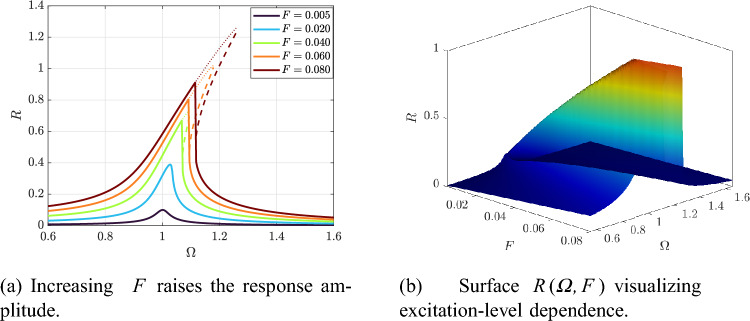
Effect of forcing amplitude *F* on the HB-predicted response at fixed $(Q,\beta )$.

**Fig. 7:**
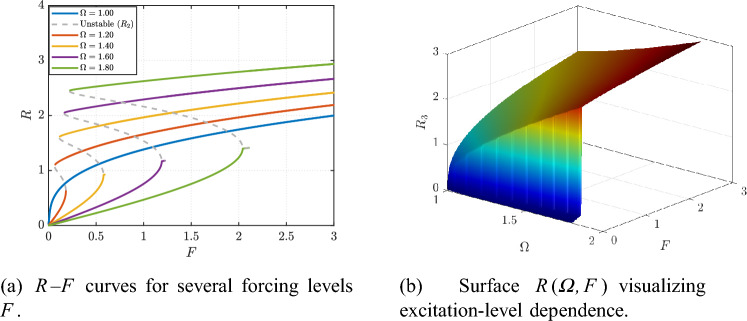
Effect of forcing amplitude *F* on the predicted response in the $(\varOmega ,f)$ plane at fixed $(Q,\beta ,\alpha )$.

The excitation level *F* further controls the attainable steady response. As shown in Fig. 6a, increasing *F* raises the resonance amplitude and enlarges the high-response region, whereas the surface in Fig. 6b provides a compact global view of how the resonant ridge grows with *F* across the $\varOmega$–*F* plane (see Fig. 6). In the alternative frequency-forcing representation, Fig. 7a reports the *R*–*f* curves for several forcing levels (with $\varOmega$ fixed per curve), and Fig. 7b summarizes the same dependence through the corresponding response surface (Fig. 7), highlighting the expansion of the multivalued/unstable region as the excitation increases.

**Fig. 8:**
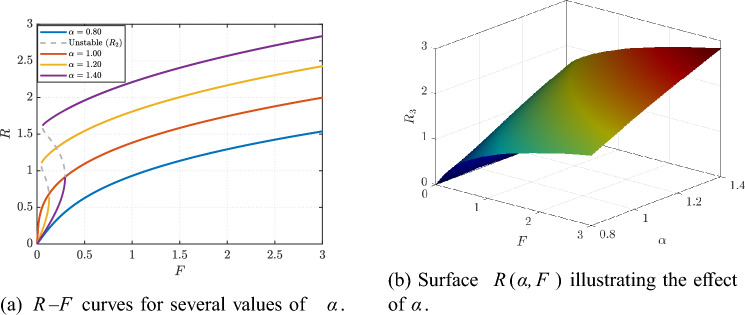
Effect of the inertia-scaling parameter $\alpha$ on the predicted response in the $(\alpha ,F)$ plane at fixed $(Q,\beta ,F)$.

Finally, Fig. 8a shows the *R*–*F* curves for several values of $\alpha$, while the surface in Fig. 8b provides a global summary of $\alpha$-driven amplitude redistribution, confirming that $\alpha$ acts as an additional tuning knob for shaping the response over the forcing range.

### Local stability of the HB periodic solution

Once the steady-state periodic response is obtained via the HB approximation, its local stability is assessed by superposing a small perturbation on the HB solution as

28 $$\begin{aligned} y(x)=y_0(x)+y_1(x). \end{aligned}$$

Substituting Eq. (28) into Eq. (19), using the fact that $y_0$ satisfies Eq. (19), and linearizing the resulting equation in $y_1$ yields the variational equation

29 $$\begin{aligned} \bigl (1+h\cos (\varOmega x)\bigr )\,y_1''+\frac{Q}{\alpha }y_1' +\left( \frac{1}{\alpha ^{2}}+\frac{3\beta }{\alpha ^{2}}y_0^{2}(x)\right) y_1=0. \end{aligned}$$

Equation (29) has $T=2\pi /\varOmega$-periodic coefficients. According to Floquet theory^36,37^, it admits solutions in the form

30 $$\begin{aligned} y_1(x)=e^{\gamma x}\rho (x), \end{aligned}$$

where $\gamma$ is the Floquet characteristic exponent and $\rho (x)$ is a *T*-periodic function, $\rho (x+T)=\rho (x)$.

Inserting Eq. (30) into Eq. (29) yields

31 $$\begin{aligned} \bigl (1+h\cos (\varOmega x)\bigr )\bigl (\rho ''+2\gamma \rho '+\gamma ^{2}\rho \bigr ) +\frac{Q}{\alpha }\bigl (\rho '+\gamma \rho \bigr ) +\left( \frac{1}{\alpha ^{2}}+\frac{3\beta }{\alpha ^{2}}y_0^{2}(x)\right) \rho =0, \end{aligned}$$

where $c=\frac{Q}{\alpha }$ will be used for compactness.

Using the HB solution with the DC component,

32 $$\begin{aligned} y_0(x)=A_0+R\cos (\varOmega x-\vartheta ),\qquad \vartheta =\tan ^{-1}\left( \frac{B_1}{A_1}\right) , \end{aligned}$$

we obtain

33 $$\begin{aligned} \begin{aligned} y_0^2(x)&=\left( A_0+R\cos (\varOmega x-\vartheta )\right) ^2\\&=\Bigl (A_0^2+\frac{R^2}{2}\Bigr ) +2A_0R\cos (\varOmega x-\vartheta ) +\frac{R^2}{2}\cos \bigl (2\varOmega x-2\vartheta \bigr )\\&=\Bigl (A_0^2+\frac{R^2}{2}\Bigr ) +2A_0R\Bigl [\cos \vartheta \cos (\varOmega x)+\sin \vartheta \sin (\varOmega x)\Bigr ]\\&\quad +\frac{R^2}{2}\Bigl [\cos (2\vartheta )\cos (2\varOmega x)+\sin (2\vartheta )\sin (2\varOmega x)\Bigr ]. \end{aligned} \end{aligned}$$

Accordingly, define the auxiliary coefficients

34 $$\begin{aligned} \begin{aligned} K_0&= \frac{1}{\alpha ^2}+\frac{3\beta }{\alpha ^2}\Bigl (A_0^2+\frac{R^2}{2}\Bigr ),\\ K_{1c}&= \frac{6\beta }{\alpha ^2}A_0R\cos \vartheta ,\qquad K_{1s} = \frac{6\beta }{\alpha ^2}A_0R\sin \vartheta ,\\ K_{2c}&= \frac{3\beta }{2\alpha ^2}R^2\cos (2\vartheta ),\qquad K_{2s} = \frac{3\beta }{2\alpha ^2}R^2\sin (2\vartheta ),\\ c&=\frac{Q}{\alpha }. \end{aligned} \end{aligned}$$

With $A_0\ne 0$, the coefficient $y_0^2(x)$ contains a fundamental component at $\varOmega$; hence, to obtain a closed set under harmonic balancing, we expand $\rho (x)$ up to the second harmonic as

35 $$\begin{aligned} \rho (x)\approx G_0+G_1\cos (\varOmega x)+H_1\sin (\varOmega x) +G_2\cos (2\varOmega x)+H_2\sin (2\varOmega x). \end{aligned}$$

Substituting Eq. (35) into Eq. (31) and comparing the coefficients of 1, $\cos (\varOmega x)$, $\sin (\varOmega x)$, $\cos (2\varOmega x)$, and $\sin (2\varOmega x)$ leads to the matrix equation

36 $$\begin{aligned} \textbf{D}(\gamma ) \begin{bmatrix} G_0\\ G_1\\ H_1\\ G_2\\ H_2 \end{bmatrix} =\textbf{0}, \end{aligned}$$

where

37 $$\begin{aligned} \textbf{D}(\gamma )= \begin{bmatrix} D_{11} & D_{12} & D_{13} & D_{14} & D_{15}\\ D_{21} & D_{22} & D_{23} & D_{24} & D_{25}\\ D_{31} & D_{32} & D_{33} & D_{34} & D_{35}\\ D_{41} & D_{42} & D_{43} & D_{44} & D_{45}\\ D_{51} & D_{52} & D_{53} & D_{54} & D_{55} \end{bmatrix}, \end{aligned}$$

and the entries are given by

38 $$\begin{aligned} & \begin{aligned} D_{11}&= \gamma ^2+c\gamma +K_0, \\ D_{12}&= \frac{h}{2}\bigl (\gamma ^2-\varOmega ^2\bigr )+\frac{1}{2}K_{1c},&D_{13}&= h\gamma \varOmega +\frac{1}{2}K_{1s}, \\ D_{14}&= \frac{1}{2}K_{2c},&D_{15}&= \frac{1}{2}K_{2s}. \end{aligned} \end{aligned}$$

39 $$\begin{aligned} & \begin{aligned} D_{21}&= h\gamma ^2+K_{1c}, \\ D_{22}&= \bigl (\gamma ^2-\varOmega ^2\bigr )+c\gamma +K_0+\frac{1}{2}K_{2c}, \\ D_{23}&= 2\gamma \varOmega +c\varOmega +\frac{1}{2}K_{2s}, \\ D_{24}&= \frac{h}{2}\bigl (\gamma ^2-4\varOmega ^2\bigr )+\frac{1}{2}K_{1c}, \\ D_{25}&= 2h\gamma \varOmega +\frac{1}{2}K_{1s}. \end{aligned} \end{aligned}$$

40 $$\begin{aligned} & \begin{aligned} D_{31}&= K_{1s}, \\ D_{32}&= -2\gamma \varOmega -c\varOmega +\frac{1}{2}K_{2s}, \\ D_{33}&= \bigl (\gamma ^2-\varOmega ^2\bigr )+c\gamma +K_0-\frac{1}{2}K_{2c}, \\ D_{34}&= -2h\gamma \varOmega -\frac{1}{2}K_{1s}, \\ D_{35}&= \frac{h}{2}\bigl (\gamma ^2-4\varOmega ^2\bigr )+\frac{1}{2}K_{1c}. \end{aligned} \end{aligned}$$

41 $$\begin{aligned} & \begin{aligned} D_{41}&= K_{2c}, \\ D_{42}&= \frac{h}{2}\bigl (\gamma ^2-\varOmega ^2\bigr )+\frac{1}{2}K_{1c},&D_{43}&= h\gamma \varOmega -\frac{1}{2}K_{1s}, \\ D_{44}&= \bigl (\gamma ^2-4\varOmega ^2\bigr )+c\gamma +K_0,&D_{45}&= 4\gamma \varOmega +2c\varOmega . \end{aligned} \end{aligned}$$

42 $$\begin{aligned} & \begin{aligned} D_{51}&= K_{2s}, \\ D_{52}&= -h\gamma \varOmega +\frac{1}{2}K_{1s},&D_{53}&= \frac{h}{2}\bigl (\gamma ^2-\varOmega ^2\bigr )+\frac{1}{2}K_{1c}, \\ D_{54}&= -4\gamma \varOmega -2c\varOmega ,&D_{55}&= \bigl (\gamma ^2-4\varOmega ^2\bigr )+c\gamma +K_0. \end{aligned} \end{aligned}$$

Non-trivial solutions of Eq. (36) exist if $\det \textbf{D}(\gamma )=0$, which yields the Floquet characteristic exponents $\gamma$. If all Floquet exponents satisfy $\Re (\gamma )<0$, then the HB periodic solution $y_0(x)$ is asymptotically stable; otherwise, it is unstable. In practice, the Floquet multipliers are $\lambda =\exp (\gamma T)$, and stability is equivalent to $|\lambda |<1$ for all multipliers.

**Fig. 9:**
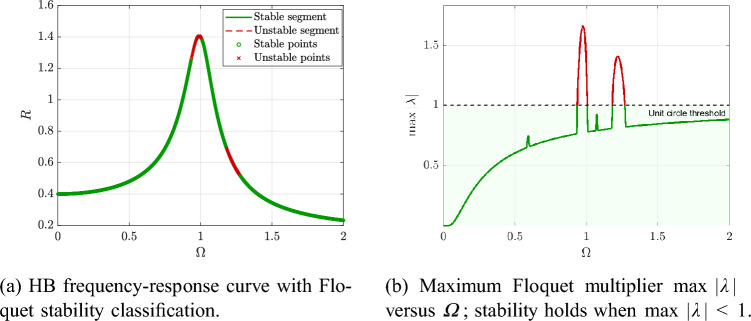
Floquet-based local stability of the HB periodic solution for $\alpha =1$, $Q=0.08$, $\beta =1$, $h=0.1$, and $\varOmega \in [0,2]$. Stable segments satisfy $\max |\lambda |<1$; otherwise the HB periodic orbit is unstable.

For clarity, Fig. 9a shows the HB frequency-response curve with Floquet-based stability classification, while Fig. 9b plots the stability indicator $\max |\lambda |$ versus $\varOmega$ together with the unit-circle threshold. Local stability holds when $\max |\lambda |<1$; once $\max |\lambda |$ crosses unity, the corresponding segment of the HB branch loses stability and is marked as unstable, providing a direct verification of the first-harmonic HB periodic solution.

## Multiple scales analysis

The method of multiple scales (MMS) is developed for the small-saliency regime of the governing Eq. (19), where the inertia-modulation depth *h* is physically small ($0<h\ll 1$)^38,39^. We assume that the external forcing, viscous damping, and cubic stiffness are weak and of the same asymptotic order as the modulation effect. Hence, we adopt the consistent ordering

43 $$\begin{aligned} F = h f, \qquad Q = h Q_0, \qquad \beta = h \beta _0, \end{aligned}$$

Under this scaling, the forcing, damping, and cubic nonlinearity enter at $\mathcal {O}(h)$, while the parametric modulation enters through the factor $(1+h\cos (\varOmega x))$ in Eq. (19). Physically, $h\ll 1$ represents a small modulation of the effective inertia, as encountered in lightly perturbed electromechanical resonators where the inertia variation over one cycle is limited to a few percent. In the operating regime of interest (near resonance with moderate response levels), dissipation is weak and the external forcing is tuned to excite observable oscillations without driving the system into a strongly nonlinear, multi-harmonic response; consequently, the damping, forcing, and cubic stiffness act as perturbative corrections to the dominant linear resonance and naturally appear at the same asymptotic order. We work directly with Eq. (19) and define $\delta =\frac{1}{\alpha }$ as the linearized natural frequency used in the near-resonance expansions. We emphasize that this ordering underpins the perturbation analysis; larger excitation levels are explored numerically in Section 'Bifurcation and chaotic response' beyond the formal validity of the weak-ordering assumption.

### Perturbation expansion

Introduce multiple time scales

44 $$\begin{aligned} X_n = h^n x, \qquad n=0,1,2,\dots , \end{aligned}$$

with $X_0$ the fast time and $X_1,X_2,\dots$ the slow times. Derivatives expand as

45 $$\begin{aligned} \frac{d}{dx}&= D_0 + h D_1 + h^2 D_2 + \cdots , \end{aligned}$$

46 $$\begin{aligned} \frac{d^2}{dx^2}&= D_0^2 + 2h D_0 D_1 + h^2\bigl (D_1^2 + 2D_0 D_2\bigr ) + \cdots , \end{aligned}$$

and the solution is sought in the series

47 $$\begin{aligned} y(x,h) = y_0(X_0,X_1,X_2) + h y_1(X_0,X_1,X_2) + h^2 y_2(X_0,X_1,X_2) + \cdots . \end{aligned}$$

Substituting Eq. (47) into Eq. (19) under the above ordering and collecting like powers of *h* yields

48 $$\begin{aligned} \mathcal {O}(1):&\quad D_0^2 y_0 + \delta ^2 y_0 = 0, \end{aligned}$$

49 $$\begin{aligned} \mathcal {O}(h):&\quad D_0^2 y_1 + \delta ^2 y_1 = -2D_0 D_1 y_0 - D_0^2 y_0 \cos (\varOmega X_0) \nonumber \\&\qquad - Q_0 \delta D_0 y_0 - \beta _0 \delta ^2 y_0^3 + f\cos (\varOmega X_0), \end{aligned}$$

50 $$\begin{aligned} \mathcal {O}(h^2):&\quad D_0^2 y_2 + \delta ^2 y_2 = -2D_0 D_1 y_1 - (D_1^2+2D_0 D_2)y_0 \nonumber \\&\qquad - D_0^2 y_1 \cos (\varOmega X_0) - 2D_0 D_1 y_0 \cos (\varOmega X_0) \nonumber \\&\qquad - Q_0 \delta D_0 y_1 - Q_0 \delta D_1 y_0 - 3\beta _0 \delta ^2 y_0^2 y_1. \end{aligned}$$

At leading order, Eq. (48) admits the harmonic solution

51 $$\begin{aligned} y_0(X_0,X_1,X_2) = \mathcal {R}(X_1,X_2)\,e^{i\delta X_0} + \text {c.c.}, \end{aligned}$$

where $\mathcal {R}(X_1,X_2)$ is a slowly varying complex envelope and $\text {c.c.}$ denotes the complex conjugate. Substituting the leading-order solution Eq. (51) into the $\mathcal {O}(h)$ Eq. (49) yields

52 $$\begin{aligned} \begin{aligned} D_0^2 y_1 + \delta ^2 y_1 =&\left[ -2i \delta D_1 \mathcal {R} \;-\; i Q_0 \delta ^2 \mathcal {R} \;-\; 3 \beta _0 \delta ^2 \mathcal {R}^2 \overline{\mathcal {R}} \right] e^{i \delta X_0} \\&\;+\; \frac{\delta ^{2}}{2}\,\mathcal {R}\, e^{i(\delta + \varOmega )X_0} \;+\; \frac{\delta ^{2}}{2}\,\mathcal {R}\, e^{i(\delta - \varOmega )X_0} \\&\;-\; \beta _0 \delta ^2 \mathcal {R}^3 e^{i 3\delta X_0} \;+\; \frac{f}{2}e^{i\varOmega X_0} \;+\; \text {c.c.}, \end{aligned} \end{aligned}$$

where $\overline{\mathcal {R}}$ denotes the complex conjugate of $\mathcal {R}$. Resonant forcing terms proportional to $\exp (\pm i\delta X_0)$ in Eq. (52) would generate secular contributions in $y_1$ of the form $X_0 e^{\pm i\delta X_0}$, thereby destroying the uniform validity of the perturbation expansion. Hence, bounded periodic solutions require the solvability condition: the coefficient of $e^{i\delta X_0}$ in Eq. (52) must vanish. This condition yields the $\mathcal {O}(h)$ modulation Landau equation governing $\mathcal {R}(X_1,X_2)$.

### Non-resonant response

When $\delta$ is far from the external frequency $\varOmega$, the solvability condition at $\mathcal {O}(h)$ from Eq. (49) gives

53 $$\begin{aligned} D_1 \mathcal {R} = -\tfrac{1}{2} Q_0 \delta \mathcal {R} + i\tfrac{3}{2}\beta _0 \delta \mathcal {R}^2 \overline{\mathcal {R}}, \end{aligned}$$

which implies exponential decay of the envelope amplitude and a cubic-induced phase drift. Writing

54 $$\begin{aligned} \mathcal {R}=\eta e^{i\mu }, \end{aligned}$$

leads to

55 $$\begin{aligned} \frac{d\eta }{dX_1} = -\tfrac{1}{2} Q_0 \delta \eta , \qquad \frac{d\mu }{dX_1} = \tfrac{3}{2}\beta _0 \delta \eta ^2. \end{aligned}$$

and hence

56 $$\begin{aligned} \eta (X_1) = \eta _0 \exp \left( -\tfrac{1}{2}Q_0 \delta X_1\right) ,\qquad \mu (X_1) = \mu _0 + \frac{3\beta _0 \eta _0^2}{2Q_0}\bigl (1-e^{-Q_0 \delta X_1}\bigr ). \end{aligned}$$

**Fig. 10:**
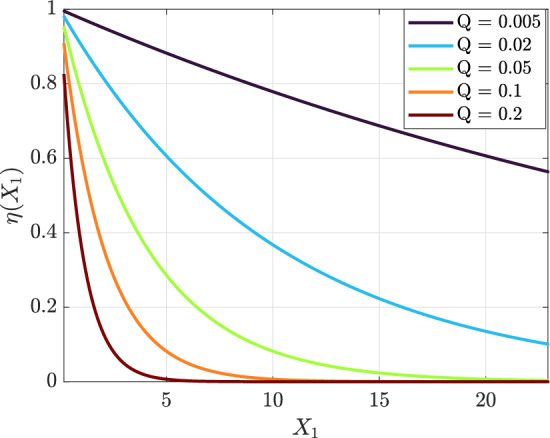
Non-resonant case: exponential decay of the envelope $\eta (X_1)$ under weak damping, with a nonlinear phase drift due to the cubic term.

The resulting envelope decay and phase drift are illustrated in Fig. 10.

### Parametric resonances

At $\mathcal {O}(h^2)$, the inertia modulation generates near-resonant interaction terms (e.g., $\exp [\pm i(\delta -2\varOmega )X_0]$ and $\exp [\pm i(2\delta -\varOmega )X_0]$) which must be eliminated by appropriate solvability conditions. This yields parametric modulation equations governing the slow evolution of the complex envelope and, consequently, the steady frequency-response curves. Since the existence of a steady response does not guarantee its observability, the stability analysis of the extracted steady solutions is also performed by linearizing the corresponding slow-flow about the fixed points. Two parametric resonance cases are of primary interest: $\delta \approx 2\varOmega$ (superharmonic) and $\delta \approx \tfrac{\varOmega }{2}$ (subharmonic).

#### Superharmonic resonance

For $$\delta \approx 2\varOmega$$, set

57 $$\begin{aligned} \delta = 2\varOmega - h^2\sigma _{1}, \end{aligned}$$

so that $\delta -2\varOmega =-h^2\sigma _1$ and hence

58 $$\begin{aligned} -i(\delta -2\varOmega )X_0 = i h^2\sigma _1 X_0 = i\sigma _1 X_2. \end{aligned}$$

Eliminating secular terms in Eq. (50) yields the Landau-type modulation equation

59 $$\begin{aligned} i\frac{d\mathcal {R}}{dX_{2}} + A\mathcal {R} + iC\,\mathcal {R}^2\overline{\mathcal {R}} + E\,\overline{\mathcal {R}} \exp \bigl (i2\sigma _{1} X_2\bigr ) = B\,\mathcal {R}^3\overline{\mathcal {R}}^2, \end{aligned}$$

with

60 $$\begin{aligned} A&= -\frac{1}{8}Q_{0}^{2}\delta +\frac{\delta }{32}\left[ \frac{(\delta +2\varOmega )^{2}}{\delta +\varOmega } -\frac{(\delta -2\varOmega )^{2}}{\delta -\varOmega } \right] , \end{aligned}$$

61 $$\begin{aligned} B&= \frac{33}{32}\beta _{0}^{2}\delta , \qquad C = -\frac{3}{4}Q_{0}\beta _{0}\delta , \qquad E = \frac{\delta ^{2}(\delta -\varOmega )^{2}}{4\,\varOmega \,(2\delta -\varOmega )}. \end{aligned}$$

and

62 $$\begin{aligned} \mathcal {R}=\frac{1}{2}a\,e^{ib}, \end{aligned}$$

Accordingly, defining the phase combination $\psi =2\sigma _{1}X_{2}-2b$ and separating the real and imaginary parts of Eq. (59), the modulation equations can be written in the amplitude-phase form

63 $$\begin{aligned} \frac{da}{dX_{2}}= -R_{1}a^{3}+R_{4}a\sin \psi , \qquad \frac{d\psi }{dX_{2}}= 2\sigma _{1}+R_{2}+R_{3}a^{4}+R_{4}\cos \psi , \end{aligned}$$

where

64 $$\begin{aligned} R_{1}=-\frac{C}{4},\qquad R_{2}=2A,\qquad R_{3}=\frac{B}{16},\qquad R_{4}=2E. \end{aligned}$$

(With this convention, $R_{1}>0$ for $Q_{0}>0$, ensuring a damping-like contribution in the amplitude equation.)

The steady-state motions occur when $da/dX_{2}=d\psi /dX_{2}=0$ in Eq. (63), which yields

65 $$\begin{aligned} \sin \psi _{0}=\frac{R_{1}a_{0}^{2}}{R_{4}}, \qquad \cos \psi _{0}=-\frac{2\sigma _{1}+R_{2}+R_{3}a_{0}^{4}}{R_{4}}. \end{aligned}$$

Eliminating $\psi _{0}$ from Eq. (65) recovers the steady frequency-response relation

66 $$\begin{aligned} (R_1 a^2)^2 + \bigl (2\sigma _{1} + R_2 + R_3 a^4\bigr )^2 - R_4^2 = 0. \end{aligned}$$

To examine the stability properties of the steady-state solutions, we superpose small perturbations about $(a_{0},\psi _{0})$ and linearize the resulting system. The eigenvalues of the corresponding Jacobian matrix satisfy

67 $$\begin{aligned} \lambda ^{2}+\mathcal {T}\lambda +\mathcal {D}=0, \end{aligned}$$

where $\mathcal {T}=-\textrm{tr}(J)$ and $\mathcal {D}=\det (J)$. For the slow-flow Eq. (63), these coefficients reduce to

68 $$\begin{aligned} \mathcal {T}= & 2R_{1}a_{0}^{2}, \end{aligned}$$

69 $$\begin{aligned} \mathcal {D}= & 2R_{1}^{2}a_{0}^{4}+4R_{3}a_{0}^{4}\Bigl (2\sigma _{1}+R_{2}+R_{3}a_{0}^{4}\Bigr ). \end{aligned}$$

Hence, the steady-state superharmonic solution is locally asymptotically stable provided that $\mathcal {T}>0$ and $\mathcal {D}>0$.

Finally, substituting $R_{1}=-C/4$, $R_{2}=2A$, and $R_{3}=B/16$ from Eq. (64) gives

70 $$\begin{aligned} \mathcal {T}=-\frac{C}{2}\,a_{0}^{2}, \qquad \mathcal {D} = 2a_{0}^{4}\left( \frac{C}{4}\right) ^{2} + \frac{B}{4}\,a_{0}^{4}\left( 2\sigma _{1}+2A+\frac{B}{16}a_{0}^{4}\right) , \end{aligned}$$

where *A*, *B*, *C*, *E* are as defined in Eqs. (60–61), and $a_{0}$ is determined from Eq. (66).

**Fig. 11:**
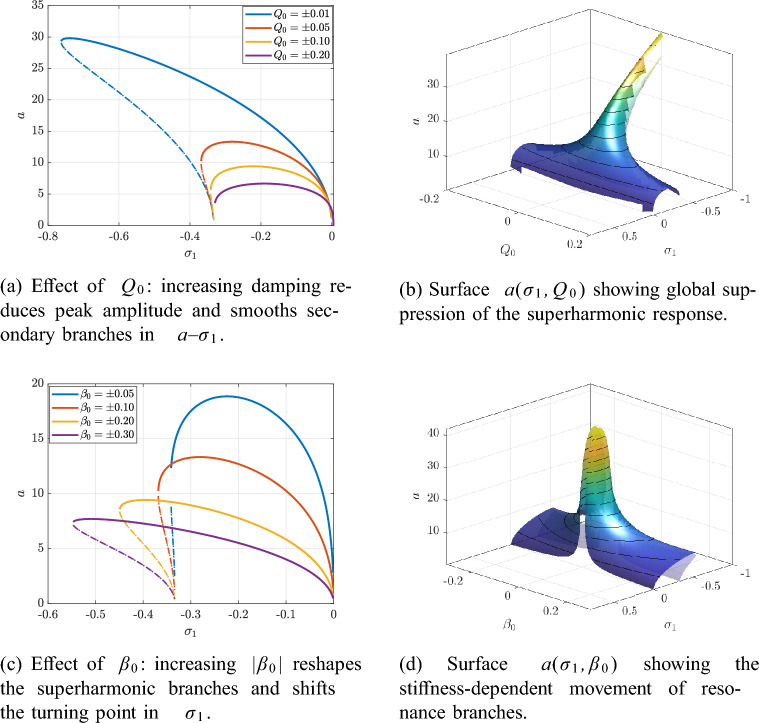
Superharmonic resonance ($\delta \approx 2\varOmega$): influence of damping $Q_0$ and cubic stiffness $\beta _0$ on the steady amplitude *a*.

The influence of damping $Q_0$ and cubic stiffness $\beta _0$ on the steady-state superharmonic response is illustrated in Fig. 11.

#### Subharmonic resonance

For $$\delta \approx \tfrac{\varOmega }{2}$$, let

71 $$\begin{aligned} \delta = \tfrac{\varOmega }{2} - \tfrac{1}{2} h^2 \sigma _2, \end{aligned}$$

so that $2\delta -\varOmega =-h^2\sigma _2$ and hence

72 $$\begin{aligned} -i(2\delta -\varOmega )X_0 = i h^2\sigma _2 X_0 = i\sigma _2 X_2. \end{aligned}$$

The resulting modulation equation is

73 $$\begin{aligned} i\frac{d\mathcal {R}}{dX_2} + A\mathcal {R} + iC\,\mathcal {R}^2\overline{\mathcal {R}} + F_p \,\overline{\mathcal {R}}^{3}\,e^{i4\sigma _2 X_2} = B\,\mathcal {R}^3 \overline{\mathcal {R}}^2, \end{aligned}$$

where *A*, *B*, *C* are as defined previously, and

74 $$\begin{aligned} F_p = \frac{(3\delta - 2\varOmega )^2}{8}\beta _0^2. \end{aligned}$$

With the parametrization Eq. (62), i.e. $\mathcal {R}=\tfrac{1}{2} a e^{ib}$, define the phase combination

75 $$\begin{aligned} \psi = 4\sigma _{2}X_{2}-4b. \end{aligned}$$

Separating the real and imaginary parts of Eq. (73) yields the amplitude-phase system

76 $$\begin{aligned} \frac{da}{dX_{2}}= -S_{1}a^{3}+S_{5}a^{3}\sin \psi , \qquad \frac{d\psi }{dX_{2}}= 4\sigma _{2}+S_{2}+S_{3}a^{4}+S_{5}a^{2}\cos \psi , \end{aligned}$$

where

77 $$\begin{aligned} S_{1}=-\frac{C}{4},\qquad S_{2}=4A,\qquad S_{3}=\frac{B}{16},\qquad S_{5}=2F_p. \end{aligned}$$

The steady-state motions occur when $da/dX_{2}=d\psi /dX_{2}=0$ in Eq. (76), which yields

78 $$\begin{aligned} \sin \psi _{0}=\frac{S_{1}}{S_{5}}, \qquad \cos \psi _{0}=-\frac{4\sigma _{2}+S_{2}+S_{3}a_{0}^{4}}{S_{5}a_{0}^{2}}. \end{aligned}$$

Eliminating $\psi _{0}$ from Eq. (78) recovers the steady frequency-response relation

79 $$\begin{aligned} (S_1 a^2)^2 - S_5^2 a^4 + \Bigl (4\sigma _{2} + S_2 + S_3 a^4\Bigr )^{2} = 0. \end{aligned}$$

To examine the stability properties of the steady-state solutions, we superpose small perturbations about $(a_{0},\psi _{0})$ and linearize the resulting system. The eigenvalues of the corresponding Jacobian matrix satisfy Eq. (67). For the slow-flow Eq. (76), these coefficients reduce to

80 $$\begin{aligned} \mathcal {T}= & 2S_{1}a_{0}^{2}, \end{aligned}$$

81 $$\begin{aligned} \mathcal {D}= & 2S_{1}^{2}a_{0}^{4} + 4S_{3}a_{0}^{4}\Bigl (4\sigma _{2}+S_{2}+S_{3}a_{0}^{4}\Bigr ). \end{aligned}$$

Hence, the steady-state subharmonic solution is locally asymptotically stable provided that $\mathcal {T}>0$ and $\mathcal {D}>0$.

Finally, substituting $S_{1}=-C/4$, $S_{2}=4A$, and $S_{3}=B/16$ from Eq. (77) gives

82 $$\begin{aligned} \mathcal {T}=-\frac{C}{2}\,a_{0}^{2}, \qquad \mathcal {D} = 2a_{0}^{4}\left( \frac{C}{4}\right) ^{2} + \frac{B}{4}\,a_{0}^{4}\left( 4\sigma _{2}+4A+\frac{B}{16}a_{0}^{4}\right) , \end{aligned}$$

where $a_{0}$ is determined from the frequency-response relation (79).

**Fig. 12:**
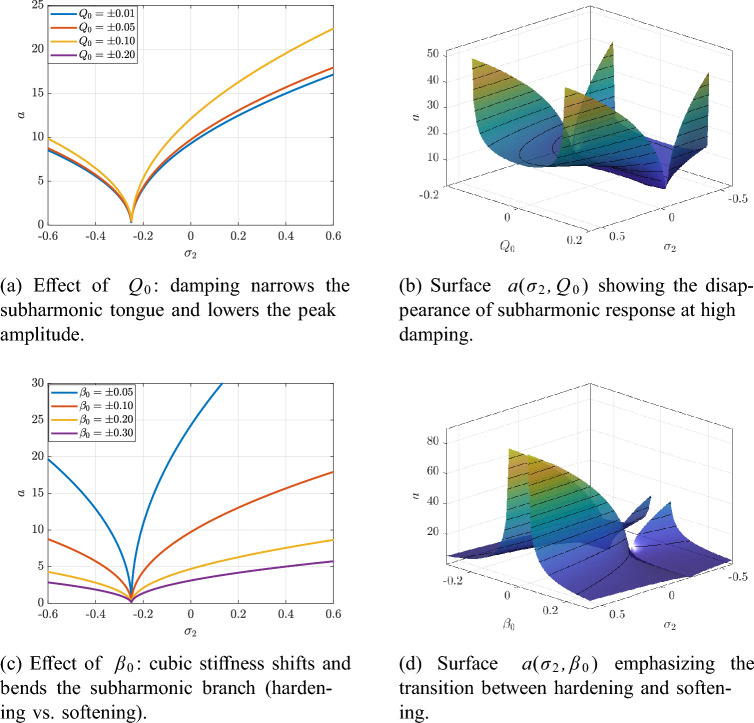
Subharmonic resonance ($\delta \approx \varOmega /2$): dependence of the steady amplitude *a* on damping $Q_0$ and cubic stiffness $\beta _0$.

The influence of damping $Q_0$ and cubic stiffness $\beta _0$ on the steady-state subharmonic response is illustrated in Fig. 12.

### Primary resonance

When $\varOmega$ is close to $\delta$, set

83 $$\begin{aligned} \varOmega = \delta + h\sigma _{p}, \end{aligned}$$

so that secular terms at $\mathcal {O}(h)$ are removed, yielding the slow-flow

84 $$\begin{aligned} -2 i \delta D_1 \mathcal {R} - i Q_0 \delta ^2 \mathcal {R} - 3 \beta _0 \delta ^2 |\mathcal {R}|^2 \mathcal {R} + \tfrac{f}{2} e^{i \sigma _p X_1} = 0, \end{aligned}$$

Using Eq. (62) gives

85 $$\begin{aligned} \frac{da}{dX_1}&= -\tfrac{1}{2} Q_0 \delta \, a + \tfrac{f}{2\delta }\sin b, \end{aligned}$$

86 $$\begin{aligned} \frac{db}{dX_1}&= \sigma _p - \tfrac{3}{8}\beta _0 \delta \, a^2 - \tfrac{f}{2\delta a}\cos b. \end{aligned}$$

Steady responses satisfy

87 $$\begin{aligned} \Bigl (-2\delta \sigma _p + \tfrac{3}{4}\beta _0 \delta ^2 a^2 \Bigr )^2 + (Q_0 \delta ^2)^2 = \Bigl (\tfrac{f}{a}\Bigr )^2. \end{aligned}$$

To establish the stability of the steady-state primary-resonance solutions, we linearize the slow-flow system Eqs. (85, 86) about its fixed points. The steady-state motions occur when $da/dX_1=db/dX_1=0$, i.e.,

88 $$\begin{aligned} \sin b_0=\frac{Q_0\delta ^{2}}{f}\,a_0, \qquad \cos b_0=\frac{2\delta a_0}{f}\left( \sigma _p-\frac{3}{8}\beta _0\delta \,a_0^2\right) , \end{aligned}$$

and eliminating $b_0$ from Eq. (88) recovers exactly the steady frequency-response relation Eq. (87).

To examine the stability properties of the steady-state solutions, we superpose small perturbations about $(a_0,b_0)$ and linearize the resulting system. The eigenvalues of the corresponding Jacobian matrix satisfy the characteristic Eq. (67). After simplification, the two coefficients reduce to

89 $$\begin{aligned} \mathcal {T}= & Q_0\delta , \end{aligned}$$

90 $$\begin{aligned} \mathcal {D}= & \left( \sigma _p-\frac{3}{8}\beta _0\delta \,a_0^2\right) \left( \sigma _p-\frac{9}{8}\beta _0\delta \,a_0^2\right) +\frac{Q_0^{2}\delta ^{2}}{4}. \end{aligned}$$

Hence, the steady-state primary-resonance solution is locally asymptotically stable provided that $\mathcal {T}>0,\quad \mathcal {D}>0$. with $a_0$ determined from Eq. (87).

**Fig. 13:**
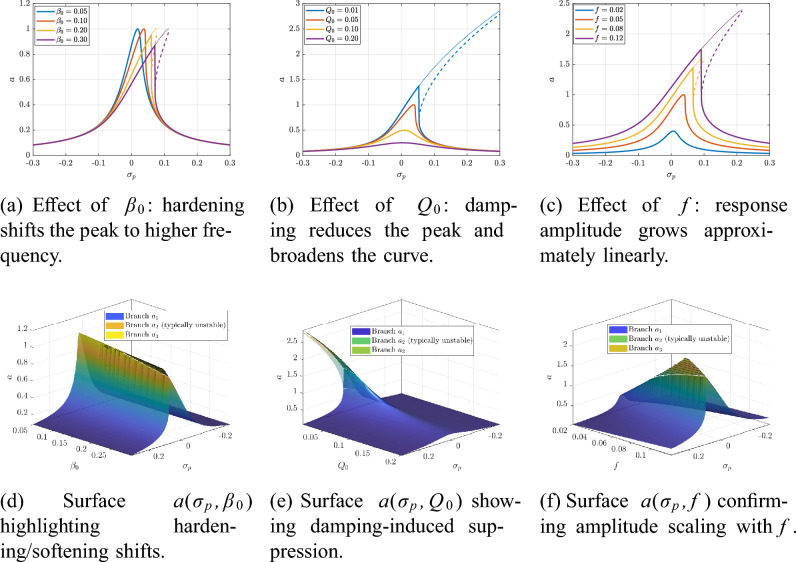
Primary resonance ($\varOmega \approx \delta$): combined effect of $\beta _0$, $Q_0$, and *f* on the steady amplitude *a*.

The combined influence of cubic stiffness $\beta _0$, damping $Q_0$, and forcing level *f* on the steady-state primary resonance response is illustrated in Fig. 13.

The MMS results show that damping suppresses resonance lobes, cubic stiffness governs hardening/softening shifts, and the forcing level controls amplitude scaling. These predictions set the stage for the fully nonlinear numerical validation reported in Section 'Bifurcation and chaotic response'.

## Comparison between Harmonic balance and multiple scales

To assess the predictive capability of the analytical steady-response approximations, we benchmark the primary-resonance solutions obtained by the harmonic balance method (HBM) and the method of multiple scales (MMS) against direct numerical time integration of Eq. (19).

Three distinct amplitude metrics are evaluated: first, the numerical reference $R_{\textrm{num}}$ is determined through long-time integration using the ODE45 solver, followed by a least-squares identification of the fundamental harmonic. Second, the HB-predicted amplitude $R_{\textrm{HB}}$ is obtained by solving the frequency-response algebraic equations given by Eq. (27). Finally, the MMS primary-resonance amplitude $a_{\textrm{MMS}}$ is derived from Eq. (87).

For each excitation frequency $\varOmega$ in a sweep centered around the natural frequency, the numerical solution is integrated over a sufficiently large number of forcing periods to remove transients. The steady response is then mapped to an amplitude through $R_{\textrm{num}}=\sqrt{a^{2}+b^{2}}$, where (*a*, *b*) are the least-squares coefficients associated with $\cos (\varOmega x)$ and $\sin (\varOmega x)$, respectively. In parallel, the HB response is evaluated by solving for the amplitude *R* via the nonlinear frequency-response relation Eq. (27). For MMS, the comparison is restricted to the asymptotically consistent near-resonance region, defined by $|\sigma _p| \le \sigma _{\textrm{valid}}$, where $\sigma _p=(\varOmega -\delta )/h$. Because Eq. (87) may admit multiple admissible real roots within this band, the MMS branch is tracked by selecting the root closest to the HB amplitude at the same $\varOmega$, thereby ensuring consistent branch identification across the sweep.

Accuracy is quantified using two complementary metrics. The root-mean-square error (RMSE) captures absolute deviations,

91 $$\begin{aligned} \textrm{RMSE}=\sqrt{\frac{1}{N}\sum _{k=1}^{N}\Bigl (R_{\textrm{pred}}(\varOmega _k)-R_{\textrm{num}}(\varOmega _k)\Bigr )^2}, \end{aligned}$$

whereas the mean absolute percentage error (MAPE) captures relative deviations,

92 $$\begin{aligned} \textrm{MAPE}=\frac{100}{N}\sum _{k=1}^{N}\frac{\bigl |R_{\textrm{pred}}(\varOmega _k)-R_{\textrm{num}}(\varOmega _k)\bigr |}{\max \bigl (R_{\textrm{num}}(\varOmega _k), \varepsilon \bigr )}. \end{aligned}$$

Here $R_{\textrm{pred}}$ denotes either $R_{\textrm{HB}}$ or $a_{\textrm{MMS}}$, and $\varepsilon$ is a small regularization threshold.

**Fig. 14:**
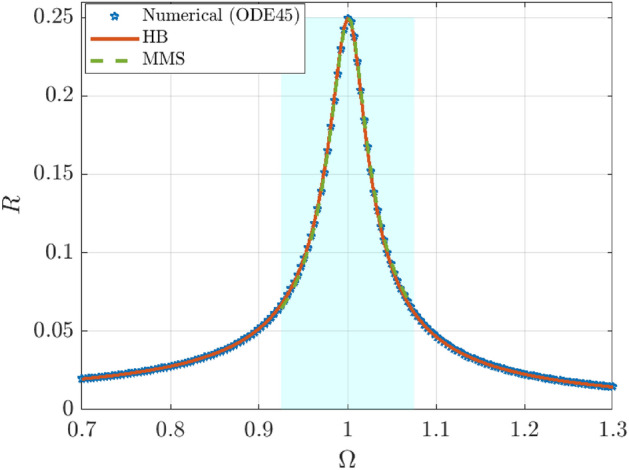
Comparison of numerical, HB, and MMS primary-resonance responses.

Figure 14 contrasts the numerical reference amplitude $R_{\textrm{num}}$ with the HB and MMS predictions across the primary-resonance sweep. The HB approximation reproduces the numerical backbone over the entire frequency range considered, indicating that the first-harmonic HB closure provides an efficient and accurate global surrogate for the steady periodic response. In contrast, MMS exhibits close agreement with the numerical solution only inside the near-resonance band, consistent with the weak-ordering assumption (43) and the local nature of the primary-resonance slow flow equations. Overall, HB and MMS offer complementary advantages: HB provides a global algebraic approximation for periodic steady states, while MMS yields a controlled local approximation with explicit parametric dependence and a transparent resonance structure.

To complement the results, Table 1 provides a quantitative comparison of the response amplitudes. The table highlights representative points across the frequency sweep, focusing on the high-amplitude resonance region.

Global accuracy is summarized by the aggregate metrics at the bottom of the table. The HB method maintains excellent consistency across the entire range (MAPE $\approx 0.75\%$). Notably, the MMS approximation becomes highly accurate near the primary resonance ($\varOmega \approx 0.99$–1.01), where the relative error drops below $0.2\%$, confirming the effectiveness of the perturbation method in predicting the peak response.

**Table 1:** Representative comparison of numerical $R_{\textrm{num}}$, $R_{\textrm{HB}}$, and $a_{\textrm{MMS}}$ amplitudes.

$\varOmega$	$R_{\textrm{num}}$	$R_{\textrm{HB}}$	$a_{\textrm{MMS}}$	$\varepsilon _{\textrm{HB}}\,(\%)$	$\varepsilon _{\textrm{MMS}}\,(\%)$
0.9250	0.0667	0.0670	0.0644	0.48	3.52
0.9663	0.1282	0.1293	0.1266	0.89	1.21
**0.9925**	**0.2299**	**0.2316**	**0.2301**	**0.78**	**0.11**
**1.0075**	**0.2376**	**0.2362**	**0.2380**	**0.57**	**0.17**
1.0750	0.0623	0.0620	0.0645	0.56	3.43
**Aggregate metrics:** HB (MAPE $0.75\%$) | MMS (MAPE $1.55\%$)					

Significant values are in bold

The data confirms that while HB provides a robust global prediction, MMS offers a compact and precise description of the system’s behavior specifically within the critical resonance band.

## Bifurcation and chaotic response

While the HB and MMS analyses provide valuable closed-form insight, their applicability depends on the assumptions and truncations adopted in the present study. In particular, the MMS results are derived under an explicit weak-ordering assumption (e.g., $F=\mathcal {O}(h)$) and therefore target slow modulation dynamics near resonance. Likewise, the HB formulation employed here is based on a first-harmonic approximation, which is primarily intended to capture the main frequency-response structure, but it is not designed to resolve broadband aperiodic motion or the detailed period-doubling cascade leading to chaos. We note that harmonic balance methods are not intrinsically limited to near-linear regimes; multi-harmonic and advanced HB variants have been successfully applied to strongly nonlinear systems (see, e.g.,^35^).

To complement these analytical predictions and characterize the strongly nonlinear regime, we carry out a high-fidelity numerical bifurcation study by systematically sweeping the forcing amplitude *F* over a wide range ($0 \le F \le 30$). This extended analysis enables us to trace the qualitative transitions in the response-from simple periodic orbits through successive period-doublings (period-1, period-2, period-4, period-8) and into fully developed chaos. By combining bifurcation diagrams, largest Lyapunov exponents, and state-space visualizations, we obtain a comprehensive characterization of the route to chaos beyond the validity of the adopted perturbation scaling and truncated harmonic approximation.

We choose the parameters as

$$\alpha = 1, \qquad \varOmega = 1, \qquad Q = 0.08, \qquad \beta = 1, \qquad h = 0.10,$$

where $\alpha$ is a dimensionless nonlinearity coefficient (set to an order-one level in the normalized model), $\varOmega =1$ defines the reference excitation frequency, $Q=0.08$ represents light damping, and $h=0.10$ is a small inertia-modulation depth consistent with $h\ll 1$.

The governing Eq. (19) is integrated using (ode45) with $\text {RelTol}=10^{-7}$, $\text {AbsTol}=10^{-9}$, and maximum step size *T*/200 where $T=2\pi /\varOmega$. The first 300 forcing periods are discarded; steady-state samples are then taken stroboscopically at $x=kT$ to form the Poincaré map.

### Remark 1

The stroboscopic sampling defines a Poincaré map that is generally two-dimensional in $(y,y')$ for the forced second-order oscillator; the plotted *y*(*kT*) is a one-dimensional projection.

**Fig. 15:**
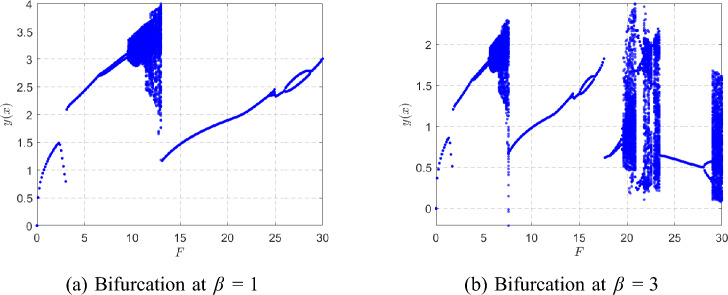
Bifurcation diagrams showing successive period-doublings and chaotic windows for $\beta =1$ and $\beta =3$.

**Fig. 16:**
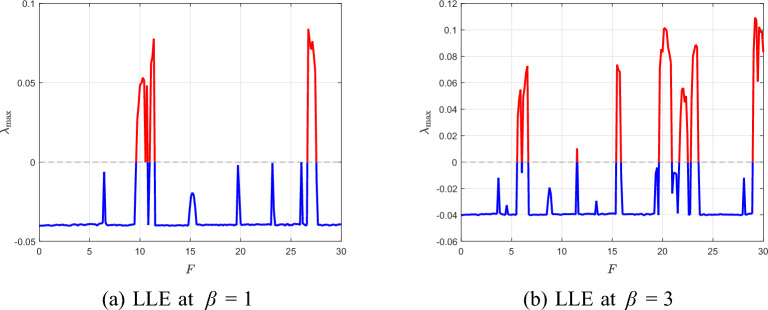
Largest Lyapunov exponent $\lambda _{\max }(F)$ confirming the transition from periodic motion ($\lambda _{\max }<0$) to chaos ($\lambda _{\max }\ge 0$).

**Fig. 17:**
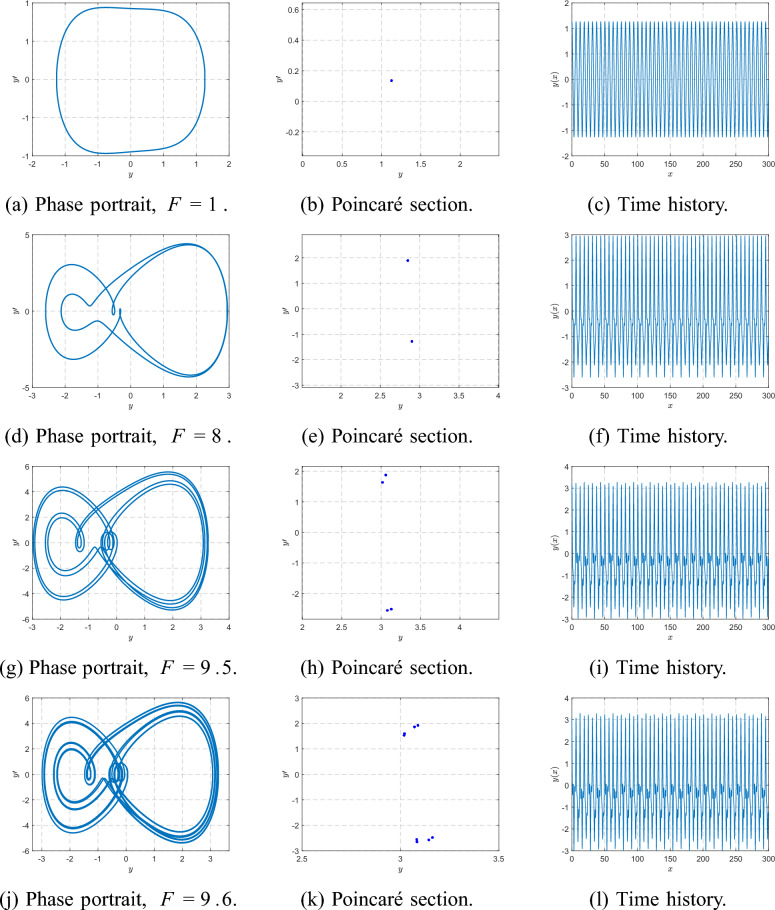
Phase portraits, Poincaré sections, and time histories at $\beta =1$ for $F=1,8,9.5,9.6$.

Figure 15 shows the global bifurcation diagrams for $\beta =1$ and $\beta =3$, revealing a period-doubling cascade (period-1 $\rightarrow$ 2 $\rightarrow$ 4 $\rightarrow$ 8) leading to chaos. At larger $\beta$, the onset of bifurcations is shifted to higher *F* and the chaotic window narrows. Figure 16 complements this view by plotting the largest Lyapunov exponent $\lambda _{\max }(F)$. Negative values correspond to periodic orbits, $\lambda _{\max }=0$ marks bifurcation onset, and positive $\lambda _{\max }$ confirms chaos. For $\beta =1$, the system starts with a single period-1 orbit at $F=1$: the phase portrait forms a closed loop, the Poincaré section shows a single point, and the time history is strictly periodic. When *F* is increased to $F=8$, the first period-doubling has already occurred and the system exhibits a period-2 orbit, with two points visible in the Poincaré section and alternating loops in the phase portrait. At $F=9.5$, a second period-doubling takes place, yielding a period-4 orbit characterized by four distinct Poincaré points and four peaks per forcing cycle in the time history. A slight increase to $F=9.6$ produces a period-8 response with eight Poincaré points, completing several stages of the period-doubling cascade. Figure 17 summarizes this progression from period-1 through period-8.

**Fig. 18:**
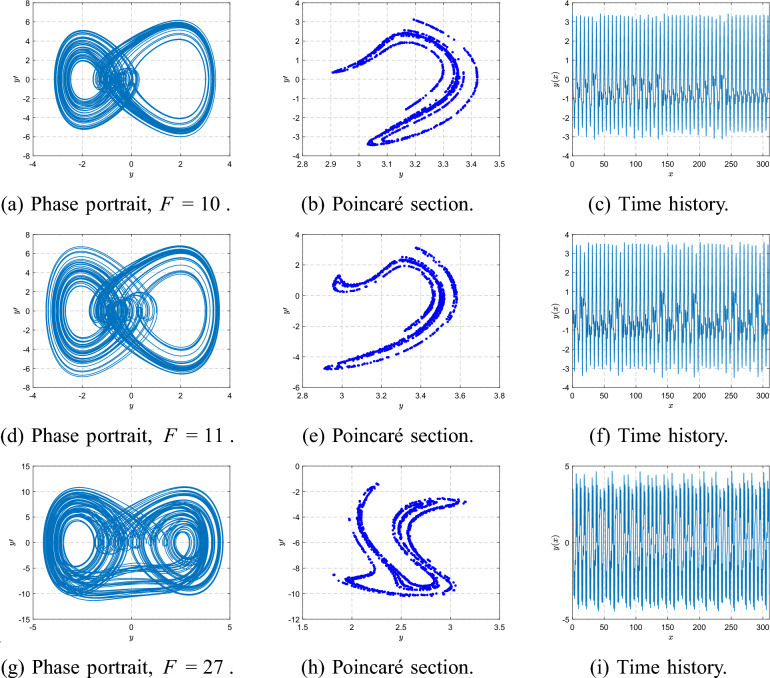
Phase portraits, Poincaré sections, and time histories at $\beta =1$ for $F=10,11,27$.

For higher forcing amplitudes ($F=10$ and $F=11$), the orbit becomes more irregular and additional bifurcations occur. By $F=27$, the system exhibits a broadband, aperiodic attractor, confirming the transition to fully developed chaos, as depicted in Fig. 18.

**Fig. 19:**
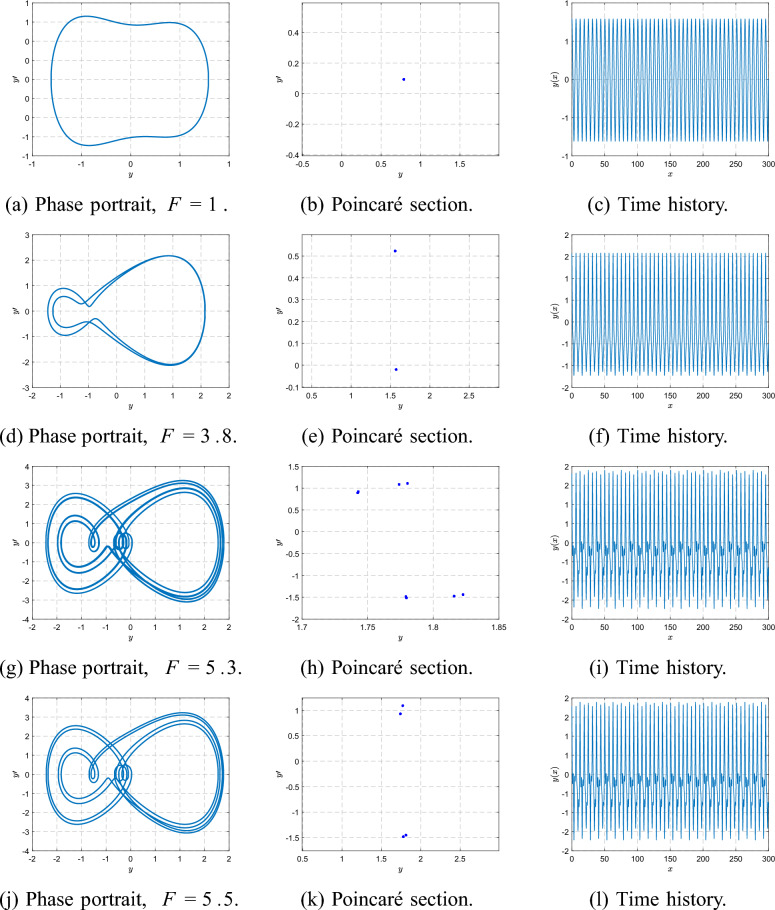
Phase portraits, Poincaré sections, and time histories at $\beta =3$ for $F=1,3.8,5.3,5.5$.

For $\beta =3$, a similar period-doubling cascade is observed but shifted toward higher *F* compared with the $\beta =1$ case. At $F=1$, the system exhibits a clean period-1 orbit: the phase trajectory is a single closed loop, the Poincaré section contains one point, and the time history is purely periodic. By $F=3.8$, the first period-doubling has occurred and the system is in a period-2 state, producing two points in the Poincaré section and alternating loops in the phase portrait. At $F=5.3$, a second doubling takes place, resulting in a period-4 orbit with four Poincaré points and four peaks per forcing cycle. A further increase to $F=5.5$ yields a period-8 orbit with eight Poincaré points, marking the last clearly periodic state before the onset of chaos. Figure  19 summarizes this evolution from period-1 through period-8.

**Fig. 20:**
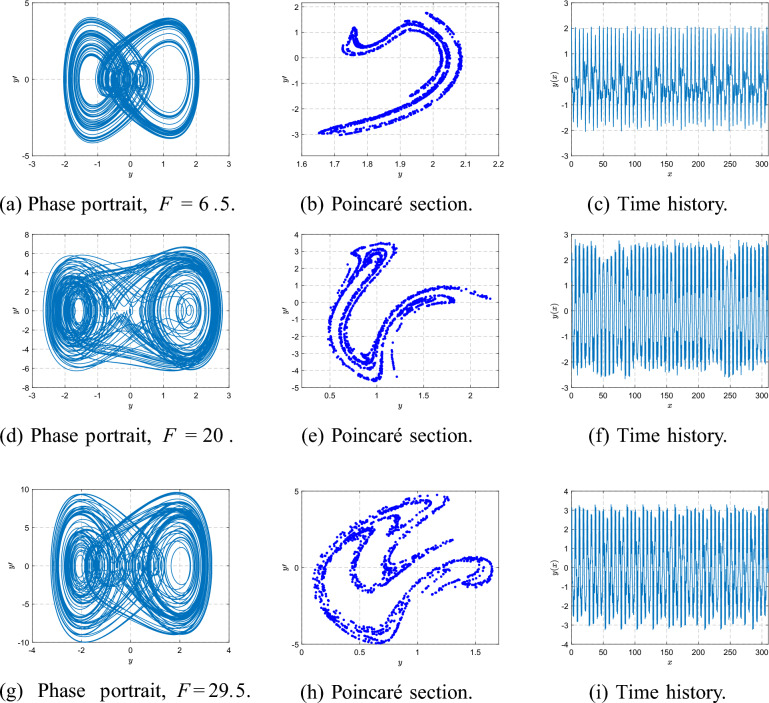
Phase portraits, Poincaré sections, and time histories at $\beta =3$ for $F=6.5,20,29.5$.

As *F* increases beyond this range, the orbit becomes irregular and transitions to a chaotic attractor. At $F=6.5$, the Poincaré section forms a scattered cloud of points and the phase portrait fills a broader region, indicating established chaos. For larger *F* values such as $F=20$ and $F=29.5$, the attractor thickens and becomes more complex, as shown in Fig. 20, reflecting fully developed broadband chaotic motion.

Taken together, the bifurcation diagrams, LLE curves, and full state-space plots provide a complete picture of the route to chaos. The observed period-doubling cascade and the positivity of $\lambda _{\max }$ as a criterion for chaos are consistent with classical nonlinear dynamics literature^40–42^.

## Piezoelectric energy harvesting: linear and nonlinear modeling

Piezoelectric energy harvesting enables the direct conversion of mechanical vibration energy into usable electrical energy and has become a key technique for powering autonomous sensors and low-power electronics^43^. Nonlinear vibration harvesters-particularly those exhibiting Duffing-type stiffness or parametric excitation-have been shown to widen the effective frequency bandwidth and improve power output under broadband and multi-frequency excitations^44–46^. The El Borhamy–Rashad–Sobhy Duffing-type oscillator, with its combination of parametric resonance, superharmonic responses, and period-doubling cascades, provides an attractive platform for energy harvesting over a wide range of operating conditions.

**Fig. 21:**
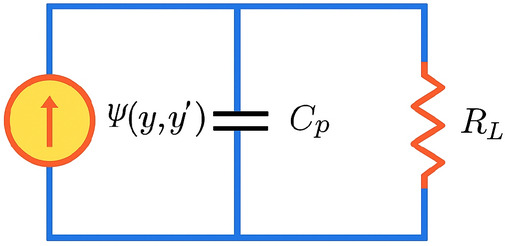
Equivalent circuit of the piezoelectric energy harvester.

The electromechanically coupled model is written as

93 $$\begin{aligned} (1+h\cos \varOmega x)\,y''+\frac{Q}{\alpha }y'+\frac{1}{\alpha ^{2}}y+\frac{\beta }{\alpha ^{2}}y^{3} =F\cos \varOmega x + \varPhi (v), \end{aligned}$$

where *v*(*x*) is the voltage across the piezoelectric branch and $\varPhi (v)$ is the feedback force acting on the mechanical structure. The electrical dynamics are represented by a parallel *R*–*C* branch (Fig. 21):

94 $$\begin{aligned} C_p v' + \frac{v}{R_L} + \varPsi (y,y') = 0. \end{aligned}$$

Physically, $\varPhi (v)$ and $\varPsi (y,y')$ describe the bidirectional piezoelectric coupling between the mechanical coordinate *y* and the electrical *R*–*C* branch. The term $\varPhi (v)$ represents the electrical back-action on the structure (voltage-to-force coupling), i.e., the voltage *v*(*x*) generates a feedback force acting on the mechanical dynamics. The term $\varPsi (y,y')$ represents the motion-induced piezoelectric current injected into the electrical branch (motion-to-current coupling); in practice it depends predominantly on the strain rate and is therefore modeled as a function of $y'$ (and, in the nonlinear case, with a weak amplitude dependence through $y^2 y'$). Hence, $\varPsi$ should be interpreted as an electromechanical coupling current term rather than as an external current source.

### Linear energy-harvesting branch

For the linear branch we set $\varPhi (v)=\varTheta v$ and $\varPsi (y,y')=\varTheta y'$. Substituting these choices into Eqs. (93–94) yields the coupled system

95 $$\begin{aligned} (1+h\cos \varOmega x)\,y''+\frac{Q}{\alpha }y'+\frac{1}{\alpha ^2}y+\frac{\beta }{\alpha ^2}y^3&= F\cos \varOmega x + \varTheta v, \end{aligned}$$

96 $$\begin{aligned} C_p v' + \frac{v}{R_L} + \varTheta y'&= 0. \end{aligned}$$

The instantaneous electrical power delivered to the load is $P(x)=v^2(x)/R_L$, and the period-averaged harvested power over one forcing period $T=2\pi /\varOmega$ is

97 $$\begin{aligned} \langle P\rangle =\frac{1}{T}\int _{x_0}^{x_0+T}\frac{v^{2}(x)}{R_L}\,dx. \end{aligned}$$

Assuming a steady-state harmonic response at the drive frequency, $y(x)=R\cos (\varOmega x-\phi )$, the electrical Eq.  (96) implies a harmonic voltage $v(x)=V\cos (\varOmega x-\phi -\psi )$. In the frequency domain,

98 $$\begin{aligned} \bigl (j\varOmega C_p + \tfrac{1}{R_L}\bigr )V + j\varOmega \varTheta R = 0, \qquad |V|=\frac{\varTheta \varOmega R}{\sqrt{(1/R_L)^2+(\varOmega C_p)^2}}. \end{aligned}$$

#### Remark 2

The RC load induces a phase lag $\psi =\tan ^{-1}(\varOmega R_L C_p)$, but $\langle P\rangle$ depends on $V^2$ and is therefore insensitive to $\psi$ once *R* is prescribed; $\psi$ matters only if $\varPhi (v)$ is used to derive a closed-loop amplitude equation for *R*.

Using $\langle v^2\rangle =V^2/2$ in Eq. (97) gives the closed-form linear harvested power

99 $$\begin{aligned} \langle P\rangle _{\textrm{lin}} =\frac{1}{2R_L}\, \frac{\varTheta ^{2}\varOmega ^{2}R^{2}}{(1/R_L)^2+(\varOmega C_p)^2}. \end{aligned}$$

This expression shows quadratic scaling with the vibration amplitude *R* and the coupling factor $\varTheta$, with a maximum near the impedance-matching condition $\varOmega R_L C_p \simeq 1$.

### Nonlinear energy-harvesting branch

To include amplitude-dependent electromechanical coupling, the same motion-induced current term $\varPsi (y,y')$ is enriched by a weak cubic contribution, yielding

100 $$\begin{aligned} C_p v' + \frac{v}{R_L} + \bigl (\theta _v + \varphi y^{2}\bigr )y' = 0, \end{aligned}$$

while $\varPhi (v)=\theta _v v$ in Eq. (93). In Eq. (100), the term $(\theta _v+\varphi y^{2})y'$ acts as a motion-induced current with an amplitude-dependent coupling. Assuming a dominant first-harmonic response $y(x)\approx a\cos (\varOmega x-\phi )$, we have $y'(x)\approx -a\varOmega \sin (\varOmega x-\phi )$ and

101 $$\begin{aligned} y^{2}y' \approx a^{2}\cos ^{2}(\varOmega x-\phi )\,y' = \frac{a^{2}}{2}\Bigl (1+\cos (2\varOmega x-2\phi )\Bigr )y'. \end{aligned}$$

Using $\sin \tau \cos 2\tau =\tfrac{1}{2}\bigl (\sin 3\tau -\sin \tau \bigr )$ with $\tau =\varOmega x-\phi$, the product $\cos (2\varOmega x-2\phi )\,y'$ contains components at $\varOmega$ and $3\varOmega$. Retaining only the fundamental component at $\varOmega$ yields

102 $$\begin{aligned} (y^{2}y')_{\varOmega } \approx \frac{a^{2}}{4}\,y', \qquad \Rightarrow \qquad (\theta _v+\varphi y^{2})y' \;\approx \; \theta _{\textrm{eff}}\,y'. \end{aligned}$$

with the effective (amplitude-dependent) coupling

103 $$\begin{aligned} \theta _{\textrm{eff}}=\theta _v+\frac{\varphi }{4}a^{2}. \end{aligned}$$

Substituting $\theta _{\textrm{eff}}$ into the linear impedance factor yields the nonlinear closed-form harvested power

104 $$\begin{aligned} \langle P\rangle _{\textrm{nl}} =\frac{1}{2R_L}\, \frac{\varOmega ^{2}\theta _{\textrm{eff}}^{2}a^{2}}{(1/R_L)^2+(\varOmega C_p)^2} = \frac{1}{2R_L}\, \frac{\varOmega ^{2}\bigl (\theta _v+\tfrac{\varphi }{4}a^{2}\bigr )^{2}a^{2}}{(1/R_L)^2+(\varOmega C_p)^2}. \end{aligned}$$

Eq. (104) predicts amplitude-dependent enhancement of harvested power, particularly near bifurcation points where *a* becomes large.

### Numerical illustration

The coupled system Eqs. (93) and (100) is numerically integrated for $\alpha =1$, $\varOmega =1/\alpha$, $Q=0.08$, $h=0.1$, $\theta _v=0.05$, $\varphi =0.02$, $C_p=0.01$, and $R_L=100$, while sweeping $F\in [0,30]$. The ordinate *y*(*kT*) denotes stroboscopic (Poincaré) samples of the displacement taken once every forcing period, i.e., $y(kT)=y(x)\big |_{x=kT}$ with $T=2\pi /\varOmega$, after discarding transients.

**Fig. 22:**
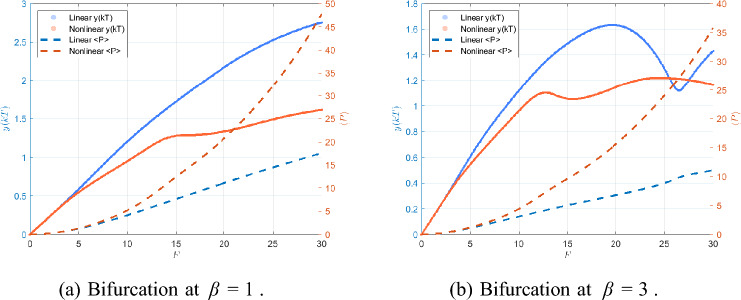
Comparison of harvested power profiles.

After discarding transients, $\langle P\rangle$ is averaged over the next 100 periods at each *F*. Figure 22 compares the harvested power profiles for $\beta =1$ and $\beta =3$, showing strong amplification in period-doubling and broadband nonlinear windows. Increasing $\beta$ shifts and reshapes these windows, suggesting that nonlinear stiffness can serve as a practical tuning knob to broaden the harvesting bandwidth^44,47^.

## Verification of harvested-power formulation

To verify the consistency of the harvested-power formulation, we compare the numerical period-averaged harvested power obtained from time-domain integration with two harmonic-based estimates: (i) the closed-form first-harmonic prediction already derived in Section 'Piezoelectric energy harvesting: linear and nonlinearmodeling' (Eq. 104), and (ii) a fundamental-fit estimate that directly validates the power definition using the extracted voltage fundamental.

For each forcing level *F*, the coupled electromechanical system Eqs. (93) and (100) is integrated over $(N_{\textrm{d}}+N_{\textrm{a}})$ forcing cycles, where the first $N_{\textrm{d}}$ cycles are discarded as transients. Over the remaining steady-state window, the numerical harvested power is computed as

105 $$\begin{aligned} \langle P\rangle _{\textrm{num}} =\frac{1}{T_{\textrm{w}}}\int _{x_0}^{x_0+T_{\textrm{w}}}\frac{v^2(x)}{R_L}\,dx, \qquad T_{\textrm{w}} = N_{\textrm{a}}\,T,\ \ T=\frac{2\pi }{\varOmega }, \end{aligned}$$

which is the discrete implementation of Eq. (97). The numerical tolerances and step-size restrictions are the same as those used in Section 'Bifurcation and chaotic response'.

Independently of the closed-form expression (104), we extract the fundamental voltage amplitude $V_1$ from the steady-state voltage using

106 $$\begin{aligned} v(x)\approx A_v\cos (\varOmega x)+B_v\sin (\varOmega x)+C_v, \qquad V_1=\sqrt{A_v^2+B_v^2}. \end{aligned}$$

If the response is quasi-harmonic at the drive frequency, then $\langle v^2\rangle \approx V_1^2/2$, hence

107 $$\begin{aligned} \langle P\rangle _{\textrm{th,fit}}=\frac{V_1^2}{2R_L}. \end{aligned}$$

Equation (107) provides a direct check of the harvested-power definition using the extracted voltage fundamental and is therefore a stronger verification than relying solely on harmonic closure in the electromechanical model.

The agreement of harmonic-based predictions degrades in strongly nonlinear windows, where the voltage waveform is no longer dominated by its fundamental component. To quantify this effect, we define the voltage non-harmonicity index

108 $$\begin{aligned} NH_v=\frac{\textrm{RMS}\left( v-v_{\textrm{fit}}\right) }{\textrm{RMS}(v)}. \end{aligned}$$

where $v_{\textrm{fit}}(x)=A_v\cos (\varOmega x)+B_v\sin (\varOmega x)+C_v$ is the fitted fundamental model in Eq. (106). We report full results over the entire sweep and also provide a verification-only subset restricted to quasi-harmonic regimes (here $NH_v<0.30$), where harmonic assumptions are expected to be meaningful.

**Fig. 23:**
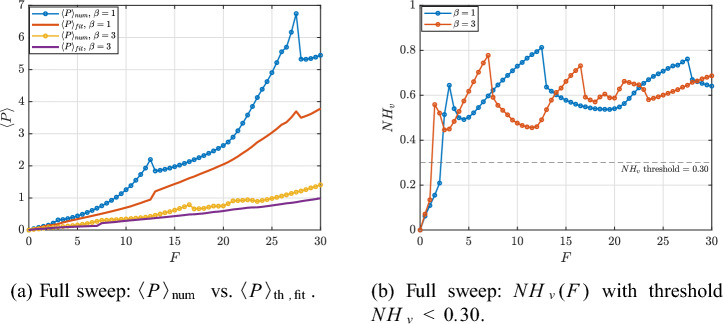
Full-sweep verification overview: (**a**) numerical vs. fit-based harvested power and (**b**) voltage non-harmonicity index, which explains the degradation of harmonic-based predictions in strongly nonlinear regimes.

Figure 23 summarizes the full-sweep verification. In Fig. 23a, the numerical average $\langle P\rangle _{\textrm{num}}$ is compared to the fit-based estimate $\langle P\rangle _{\textrm{th,fit}}=V_1^2/(2R_L)$ across $F\in [0,30]$ for $\beta =1$ and $\beta =3$. A close agreement is observed whenever the voltage remains quasi-harmonic at the drive frequency, which confirms the correctness of the numerical averaging and the harvested-power definition. Figure 23b complements this by reporting the non-harmonicity index $NH_v(F)$; as $NH_v$ increases, the steady-state voltage contains substantial non-fundamental content and harmonic-based predictions are expected to degrade.

**Fig. 24:**
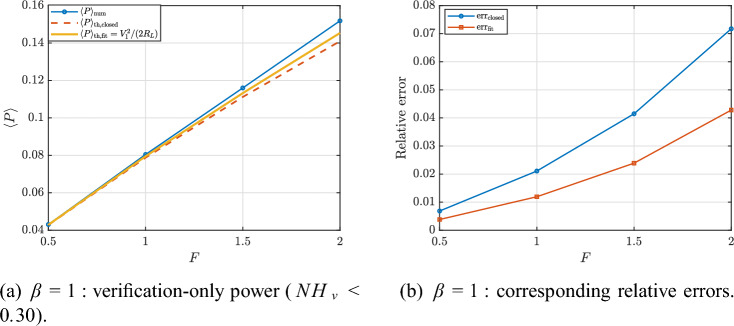
Verification-only subset ($NH_v<0.30$) for $\beta =1$: numerical power compared with the closed-form prediction [Eq. (104)] and the fit-based estimate [Eq. (107)], together with the corresponding relative errors.

**Fig. 25:**
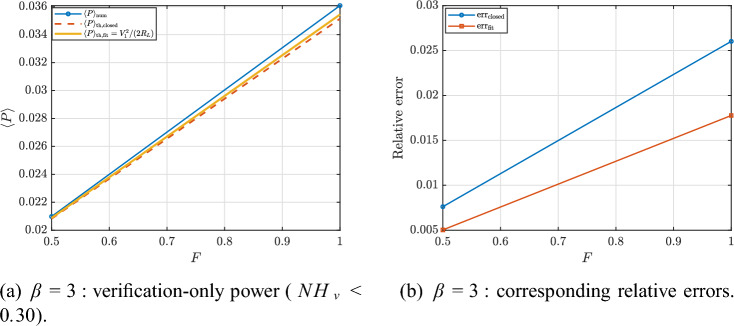
Verification-only subset ($NH_v<0.30$) for $\beta =3$: numerical power compared with the closed-form prediction [Eq. (104)] and the fit-based estimate [Eq. (107)], together with the corresponding relative errors.

To isolate the regime where harmonic reasoning is meaningful, we extract a verification-only subset defined by $NH_v<0.30$. Figures 24 and 25 report the corresponding power curves and relative errors for $\beta =1$ and $\beta =3$, respectively. In this subset, the fit-based estimate $\langle P\rangle _{\textrm{th,fit}}$ [Eq. (107)] provides the closest agreement with $\langle P\rangle _{\textrm{num}}$ because it directly validates the power definition using the extracted voltage fundamental. By contrast, the closed-form prediction [Eq. (104)] can deviate more noticeably as nonlinear distortions and higher harmonics increase, even when the response remains only moderately non-harmonic. Representative verification-only points are listed in Tables 2 and 3.

**Table 2:** Verification points for $\beta =1$ under $NH_v<0.30$.

*F*	$\langle P\rangle _{\textrm{num}}$	$\langle P\rangle _{\textrm{th,closed}}$	$\langle P\rangle _{\textrm{th,fit}}$	err$_{\textrm{closed}}$	err$_{\textrm{fit}}$	$NH_v$
	$(\times 10^{-2})$	$(\times 10^{-2})$	$(\times 10^{-2})$	$(\times 10^{-2})$	$(\times 10^{-2})$	$(\times 10^{-2})$
0.5	4.3066	4.2772	4.2902	0.6847	0.3822	6.1903
1.0	8.0456	7.8760	7.9496	2.1078	1.1924	10.9260
1.5	11.6000	11.1190	11.3230	4.1469	2.3881	15.5100
2.0	15.1850	14.0960	14.5350	7.1731	4.2788	20.9100

**Table 3:** Representative verification-only points for $\beta =3$, restricted to $NH_v < 0.30$.

*F*	$\langle P\rangle _{\textrm{num}}$	$\langle P\rangle _{\textrm{th,closed}}$	$\langle P\rangle _{\textrm{th,fit}}$	err$_{\textrm{closed}}$	err$_{\textrm{fit}}$	$NH_v$
	$(\times 10^{-2})$	$(\times 10^{-2})$	$(\times 10^{-2})$	$(\times 10^{-2})$	$(\times 10^{-2})$	$(\times 10^{-2})$
0.5	2.0982	2.0823	2.0877	0.7618	0.5037	7.1025
1.0	3.6081	3.5143	3.5440	2.6017	1.7767	13.4520

## The conclusion

This paper developed a unified analytical-numerical framework to characterize a Duffing-type oscillator with harmonically modulated inertia, starting from an energy-based electromechanical formulation and a consistent non–dimensional model that retains the inertia modulation as a multiplicative parametric factor in the highest-order term. Two complementary perturbation tools have been then combined: Harmonic Balance (HBM), providing explicit frequency-response branches and backbone relations, and the method of multiple scales (MMS), yielding amplitude-phase modulation equations that organize primary, superharmonic, and subharmonic resonances within a single small-saliency description.

Beyond classical steady-state predictions, the study quantified how inertia modulation reshapes the operating envelope of the oscillator. High-fidelity simulations, together with Poincaré sections, global bifurcation diagrams, and largest Lyapunov exponents, consistently revealed a transition scenario from periodic motion to complex dynamics via period-doubling cascades. In particular, the results identify how (i) damping reduces resonance amplitudes but preserves the underlying nonlinear resonance structure, (ii) the cubic stiffness parameter controls the hardening/softening trend and shifts the onset of multi-stability and complex responses, and (iii) periodic inertia modulation enlarges the instability regions and accelerates the emergence of period-doubling windows and chaotic-like attractors, thus narrowing stability margins compared with constant-inertia operation.

A further contribution is the integration of the nonlinear oscillator with a piezoelectric energy-harvesting branch, including an amplitude-dependent coupling description that leads to a tractable closed-form estimate of the harvested power. The numerical results show a strong correlation between large-amplitude (and, in particular, broadband nonlinear) responses and increased average harvested power. This establishes a design-relevant trade-off: inertia modulation and nonlinear stiffness can be exploited to widen the effective harvesting bandwidth and raise power levels, but at the cost of reduced stability margins and a higher likelihood of multi-stability/complex dynamics.

Overall, the proposed framework provides a reproducible benchmark that connects perturbation-based resonance analysis (HBM/MMS) with time-series diagnostics (Poincaré/Lyapunov) for inertia-modulated oscillators, and it clarifies when nonlinear resonance mechanisms can be deliberately leveraged for energy-harvesting enhancement. Future work will address stochastic/broadband excitations, targeted suppression or regulation of undesirable complex dynamics, and experimental validation on a physical prototype.

## Data Availability

The data used to support the findings of this study are available from the authors upon request.

## Declarations

### Animal research

For this research article, the authors did not undertake work that involved human participants or animals.
